# *ZFAS1:* a long noncoding RNA associated with ribosomes in breast cancer cells

**DOI:** 10.1186/s13062-016-0165-y

**Published:** 2016-11-21

**Authors:** Herah Hansji, Euphemia Y. Leung, Bruce C. Baguley, Graeme J. Finlay, David Cameron-Smith, Vandre C. Figueiredo, Marjan E. Askarian-Amiri

**Affiliations:** 1 Auckland Cancer Society Research Centre, University of Auckland, 85 Park Rd, Grafton, Auckland, 1023 New Zealand; 2 Department of Molecular Medicine and Pathology, University of Auckland, 85 Park Rd, Grafton, Auckland, 1023 New Zealand; 3 The Liggins Institute, University of Auckland, 85 Park Rd, Grafton, Auckland, 1023 New Zealand

**Keywords:** *ZFAS1*, lncRNA, Ribosome, Protein translation, Breast cancer, Small subunit of ribosome

## Abstract

**Background:**

Most of the eukaryotic genome is transcribed, yielding a complex network of transcripts including thousands of lncRNAs that generally lack protein coding potential. However, only a small percentage of these molecules has been functionally characterised, and discoveries of specific functions demonstrate layers of complexity. A large percentage of lncRNAs is located in the cytoplasm, associated with ribosomes but the function of the majority of these transcripts is unclear. The current study analyses putative mechanisms of action of the lncRNA species member *ZFAS1* that was initially discovered by microarray analysis of murine tissues undergoing mammary gland development. As developmental genes are often deregulated in cancer, here we have studied its function in breast cancer cell lines.

**Results:**

Using human breast cancer cell lines, *ZFAS1* was found to be expressed in all cell lines tested, albeit at different levels of abundance. Following subcellular fractionation, human *ZFAS1* was found in both nucleus and cytoplasm (as is the mouse orthologue) in an isoform-independent manner. Sucrose gradients based on velocity sedimentation were utilised to separate the different components of total cell lysate, and surprisingly *ZFAS1* was primarily co-localised with light polysomes. Further investigation into ribosome association through subunit dissociation studies showed that *ZFAS1* was predominantly associated with the 40S small ribosomal subunit. The expression levels of *ZFAS1* and of mRNAs encoding several ribosomal proteins that have roles in ribosome assembly, production and maturation were tightly correlated. *ZFAS1* knockdown significantly reduced RPS6 phosphorylation.

**Conclusion:**

A large number of lncRNAs associate with ribosomes but the function of the majority of these lncRNAs has not been elucidated. The association of the lncRNA *ZFAS1* with a subpopulation of ribosomes and the correlation with expression of mRNAs for ribosomal proteins suggest a ribosome-interacting mechanism pertaining to their assembly or biosynthetic activity. *ZFAS1* may represent a new class of lncRNAs which associates with ribosomes to regulate their function.

**Reviewers:**

This article was reviewed by Christine Vande Velde, Nicola Aceto and Haruhiko Siomi.

**Electronic supplementary material:**

The online version of this article (doi:10.1186/s13062-016-0165-y) contains supplementary material, which is available to authorized users.

## Background

Long non-coding RNAs (lncRNAs) comprise a large proportion of the transcribed RNA species in cells, and are responsible for a diversity of functions [1]. Early studies of lncRNAs showed that they are located in the nucleus, in which they interact with different chromatin-modifying complexes, resulting in a paradigm in which lncRNAs regulate transcription through chromatin modification [2]. The field of lncRNA research has rapidly expanded, and novel functions have been identified. LncRNAs are versatile molecules that function also in the cytoplasm where they interact with other RNA species to regulate their processing and post-transcriptional regulation, with numerous proteins to regulate their function and with ribonucleoprotein complexes to modulate translation, either of specific genes or of protein synthesis globally [3].

LncRNAs are involved in complex biological processes such as normal development and disease pathogenesis [4]. Several lncRNAs are associated with known developmental protein-coding genes, and many lncRNAs are differentially expressed during induced differentiation of embryonic stem cells [5]. Loss of function of a subset of lncRNAs involved in embryonic development leads to loss of pluripotency or commitment to differentiation programmes [6, 7]. LncRNAs have also been identified as establishing and maintaining gene expression patterns during the development of different tissues and organs, including breast tissue, in which they contribute to the differentiation and organisation of the mammary epithelium [8].

Recent studies have found that a large proportion of long non-coding RNAs, despite lacking protein-coding potential, are associated with the ribosomes [9–11]. The precise function of the majority of these transcripts interacting with ribosomes remains unknown, although those few that have been studied in detail have been found to regulate translation by associating with the polysomes during stress conditions and regulating the translation of specific mRNAs, such as *Uchl1AS*, an antisense lncRNA which regulates its protein-coding partner *UCHL1* [12]. This however is unlikely to be the main mechanism of action of ribosome-associated lncRNAs as few antisense lncRNAs colocalise with their protein-coding partners [9]. Other lncRNAs associate with ribosomes for targeted degradation, but transcriptome-wide studies of ribosome-mediated degradation have found only a few lncRNAs utilising this pathway [13]. Given the complexity of ribosomes, from their biogenesis to their synthetic function, there are many possible avenues by which lncRNAs could regulate ribosomal activity. Ribosomes have long been known to be deregulated in tumourigenesis, with a large number of tumour suppressor genes and oncogenes modifying the translational activity of ribosomes [14].

**Fig. 1:**
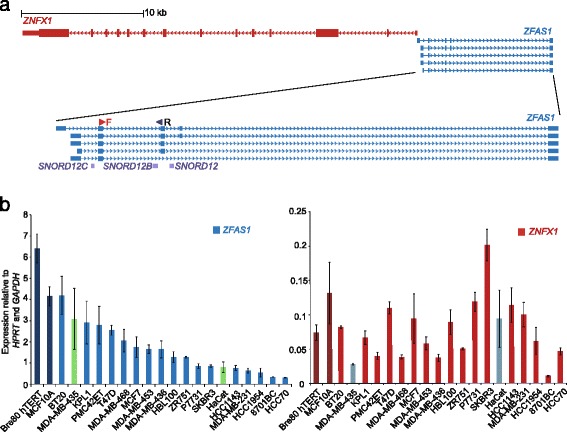
Genomic orientation of *ZFAS1* and *ZNFX1* and their expression. **a** Genomic orientation of *ZFAS1* in relation to *ZNFX1* derived from the UCSC Genome Browser genome assembly Mar 2006 (NCBI36/hg18). The enlarged figure of *ZFAS1* shows the location of the three snoRNAs in three consecutive introns. **b** Expression of *ZFAS1* (*left*) and *ZNFX1* (*right*) in cell lines relative to that of *HPRT* and *GAPDH*. Dark blue and dark red bars indicate normal breast epithelial cell lines, normal coloured bars indicate breast cancer cell lines. Green and grey coloured bars indicate non-breast cancer cell lines (MDA-MB-435 and HaCat are melanoma and keratinocyte cell lines respectively). *ZNFX1* and *ZFAS1* expression showed no correlation. Expression levels were analysed in 3 biological replicates, error bars represent SEM. Primers used for qPCR are shown in panel **a** (forward (F) and reverse (R) primers on exons 2 and 3 respectively)

Recent microarray analyses of tissue from mouse mammary glands at different stages of post-pubertal development have revealed that several lncRNAs are differentially expressed in developing mammary glands [15]. Of these lncRNAs, a previously uncharacterised lncRNA (GenBank ID AK005231) was studied, as it is differentially expressed during mouse mammary gland development and also found at the syntenic region in the human genome. This lncRNA, *Zfas1,* is located on the antisense strand of the *Znfx1* (zinc finger NFX-1-type containing) promoter region and is host to three snoRNAs. Further analysis of this lncRNA showed that it is expressed in most tissues, but showed greatest abundance in developing mammary glands [15]. In vivo, *Zfas1* was found to be restricted to the epithelial cells of the mammary gland ducts and alveoli of pregnant mice. Knockdown of *Zfas1* by siRNA in a mouse mammary epithelial cell line increased cellular differentiation significantly and to a lesser extent induced proliferation [15]. These experiments suggested that *Zfas1* plays important roles in mammary gland development. In the human genome the lncRNA antisense to *ZNFX1* showed similar structure to *Zfas1* in mouse (Fig. 1a). Given its role in mammary epithelial proliferation and differentiation, *ZFAS1* expression was compared in human invasive ductal carcinoma and in normal breast tissue, and was found to be decreased in abundance in the former [15]. These results prompted further study of the function of *ZFAS1* using human breast cancer cell lines.

According to the Mar 2006 NCBI36.1/hg18 genome assembly, at least five different isoforms of *ZFAS1* exist [16]. They vary in size from 516 to 1006 bases with exons two and five common to all isoforms. In the present study, we sought to identify different isoforms of *ZFAS1* and to investigate their cellular localisation. We confirmed that *ZFAS1* is expressed as at least five different isoforms, found in both cytoplasmic and nuclear compartments. We also found that cytoplasmic *ZFAS1* is localised primarily with 80S ribosomes and light polysomes, and ribosome dissociation studies showed that *ZFAS1* is associated with the small subunit of the ribosome. Global inhibition of ribosome activity through growth arrest and treatment with the translation inhibitor puromycin leads to an increase of *ZFAS1* content in certain cell lines. *ZFAS1* expression is strongly correlated with that of a number of mRNAs encoding ribosomal proteins involved in ribosome biogenesis, and its abundance also increases upon induced ribosome biogenesis. Knockdown of *ZFAS1* decreases the phosphorylation state of the ribosomal protein RPS6. *ZFAS1* may be involved in the regulation of the ribosome through interactions with mature ribosomes in the cytoplasm as well as through interactions with immature ribosomes in the nucleus.

## Results

### Protein-coding potential for *ZFAS1*

Alternative splicing of pre-mature RNA is an important process that increases the repertoire of mRNA isoforms. Five different isoforms (Fig. 1a) have been reported for *ZFAS1* according to the Mar 2006 NCBI36.1/hg18 genome assembly. To ensure that the human variants of *ZFAS1* are non-protein-coding as demonstrated in mice [15], predicted open reading frames generated from ExPASy for each isoform were aligned against Riboseq data derived from GWIPS-viz [17] to determine whether predicted peptides matched those identified by ribosomal occupancy (Additional file 1: Figure S1). The majority of the peaks corresponding to ribosomal occupancy overlapped with genomic regions of intron-derived snoRNAs. These peaks are a source of background RNA in profiling experiments, similar to that of *GAS5,* another lncRNA that is host to several snoRNAs as described by Ingolia et al. [18]. A peak in ribosomal occupancy was observed in exon 2 of the *ZFAS1* isoforms, which corresponded to an open reading frame (ORF) that predicted a peptide of 25 amino acids (M D F G R G S H H W T S K E A T C R H L Q P S I S Stop). A query of PeptideAtlas, a database of peptide sequences deduced from proteomic analyses [19], showed that no peptides have been identified that correspond to this particular ORF. Together, these observations led us to conclude that the human isoforms of *ZFAS1* are unlikely to encode a peptide.

### Expression of *ZFAS1* and *ZNFX1* in breast cancer: cell line and The Cancer Genome Atlas data

*ZFAS1* is expressed in mouse mammary gland tissues, and was previously found to be downregulated in human invasive ductal breast carcinoma, as compared to normal breast tissue [15]. To further analyse *ZFAS1* expression and to characterise its function, we have used breast cancer as our model system. We performed qPCR on cDNA prepared from 17 breast cancer cell lines, a keratinocyte cell line (HaCat), a melanoma cell line (MDA-MB-435) and two breast epithelial cell lines, Bre80hTert and MCF10A. Different levels of expression of *ZFAS1* were detected in these cell lines, as shown in Fig. 1b. *ZNFX1* was also expressed in these cell lines, and as with previous results [15], was approximately 25 fold less abundant than *ZFAS1* (Fig. 1b). The expression levels of *ZFAS1* and *ZNFX1* were not significantly correlated (Pearson correlation coefficient, R = −0.0021, *p* = 0.93), further suggesting that *ZFAS1* and *ZNFX1* are independently regulated (Fig. 1b). We also could not detect any significant difference in *ZFAS1* or *ZNFX1* expression between ER+ (*n* = 9) and ER- (*n* = 5) cell lines (*P* = 0.38) (data not shown). Many lncRNAs regulate the protein-coding genes in *cis*. If this was the case with *ZFAS1* and *ZNFX1*, it would be expected that the abundance of their transcripts should be related. The lack of any correlation is evidence that *cis* regulation involving this pair of genes does not apply, and provides a basis for seeking alternative *ZFAS1* activities.

We also analysed the genome-wide RNA transcript profile from TCGA (breast invasive carcinoma expression) by RNAseq data set (HiSeqV2-2015-02-24) including 1049 samples from primary breast cancers and 113 samples from normal breast tissue. The expression of *ZFAS1* was not significantly different in breast cancer patients as compared to healthy controls (*P* = 0.4941) as shown in Additional file 2: Figure S2A(i). However, *ZFAS1* expression was significantly reduced in basal (*p* = 0.0331) and HER2 (*p* = 0.0011) breast cancer subtypes (Additional file 2: Figure S2A(ii)) compared to normal breast tissue. ER+ (*n* = 601) breast tumours also displayed higher expression of *ZFAS1* compared to ER- (*n* = 179) negative breast tumours (*p* = 0.0212) (Additional file 2: Figure S2A(iii)). Our earlier publication (2011), using a limited number of samples, suggested that *ZFAS1* expression was down-regulated in breast cancer cells relative to normal breast epithelial cells. Our current study sought to investigate this finding more thoroughly, using large TCGA datasets, and found no differences between unselected neoplastic and normal breast samples. The possible subtle differences between *ZFAS1* expression in certain *subtypes* of breast cancer and normal cells (Additional file 2: Figure S2A) could reflect the large number of samples examined, and thus be of minimal clinical impact.

Additional file 2: Figure S2B displays a Kaplan-Meier plot generated from http://www.oncolnc.org of TCGA breast cancer data set. High expressers are those 50% of patients with the highest *ZFAS1* expression, and low expressers are those 50% of patients with the lowest *ZFAS1* expression. These groups do not show significant differences in survival up to 6000 days.

### *ZFAS1* isoforms are located in the cytoplasm

**Fig. 2:**
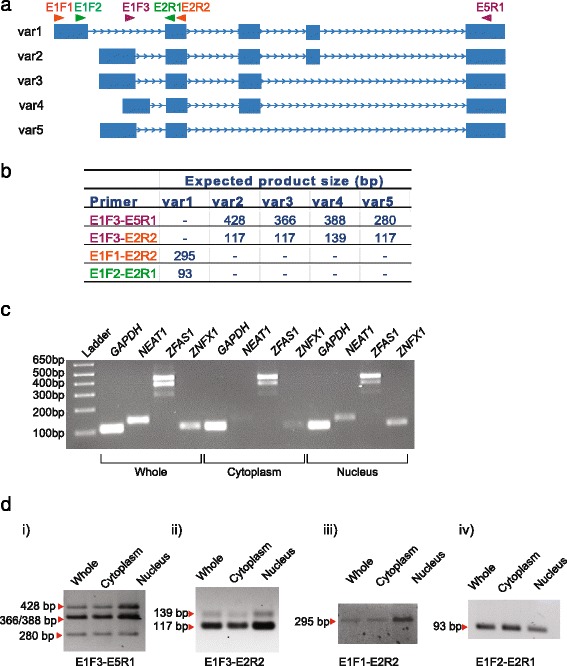
Detection of *ZFAS1* isoforms by RT-PCR and demonstration of their subcellular location. **a** Orientation of primers used in RT-PCR in relation to *ZFAS1* genomic arrangement. Primers were designed to cover all isoforms. **b** Primer pairs used to detect *ZFAS1* isoforms and the expected PCR product sizes for different isoforms. **c** Localisation of *ZFAS1* and *ZNFX1* in cellular compartments as detected by PCR in MDA-MB-468 cells. *ZFAS1* was amplified using primer set E1F3-E5R1 and shown to be present in cytoplasm and nucleus. *ZNFX1* was expressed predominantly in the nucleus. *NEAT1,* a nuclear lncRNA was used as a nuclear control; *GAPDH* was used as a positive control. **d** Expression of *ZFAS1* isoforms using different primer pairs in MDA-MB-468 cellular fractions. i) E1F3-E5R1 amplified all isoforms except var1, allowing detection of var2-5. (ii) E1F3-E2R2 amplified the first exons of var2-5 in extracts of both cytoplasm and nucleus. (iii) To identify var1, PCR was performed in the first and second exons using primer set E1F1-E2R2, and (iv) verified by internal PCR using primers E1F2-E2R1, yielding a 93 bp product in both cytoplasm and nucleus

Previous experiments in mice showed that *Zfas1* was found in both cytoplasm and nucleus, whereas *Znfx1* mRNA was restricted to the nucleus [15]. Since cellular location will dictate function of lncRNA, cellular fractionation was performed to identify the subcellular localisation of *ZFAS1* in MDA-MB-468 and MDA-MB-231 breast cancer cells. Cell lysates were separated into cytoplasmic and nuclear fractions, total RNA extracted and analysed by RT-PCR. *ZFAS1* is present as 5 isoforms (Figs. 1a and 2a) and primers were designed to amplify all isoforms while allowing differentiation of these isoforms through product size (Fig. 2b). For the purpose of validating the effectiveness of the fractionation procedure, we used *NEAT1*, a nuclear lncRNA, as a nuclear marker, and *GAPDH* as a positive control as it is found in both the cytoplasm and nucleus (Fig. 2c). As indicated for MDA-MB-468 cell extracts, *ZFAS1* was found in both the cytoplasm and nucleus, indicating that *ZFAS1* is not restricted to a specific cellular compartment. *ZNFX1* on the other hand is enriched in the nucleus (Fig. 2c) as previously reported [15]. All the isoforms of *ZFAS1* identified were present in both the cytoplasm and the nucleus; therefore the functions of the isoforms are not distinguishable by compartmentalisation (Fig. 2d). No difference between the two cell lines was observed.

### Identification of a *ZFAS1* binding partner in cytoplasm

The functions of numerous lncRNAs have been deduced by identifying their binding partners. To infer possible *ZFAS1* functions, we explored whether *ZFAS1* was associated with macromolecular complexes. Total cell lysate was separated on a sucrose gradient by the principle of velocity sedimentation. If *ZFAS1* was associated with large structures such as ribosomes or spliceosomes, it would migrate further down the sucrose gradient. Conversely, if *ZFAS1* was associated with small protein complexes, it would remain near the top of the gradient.

**Fig. 3:**
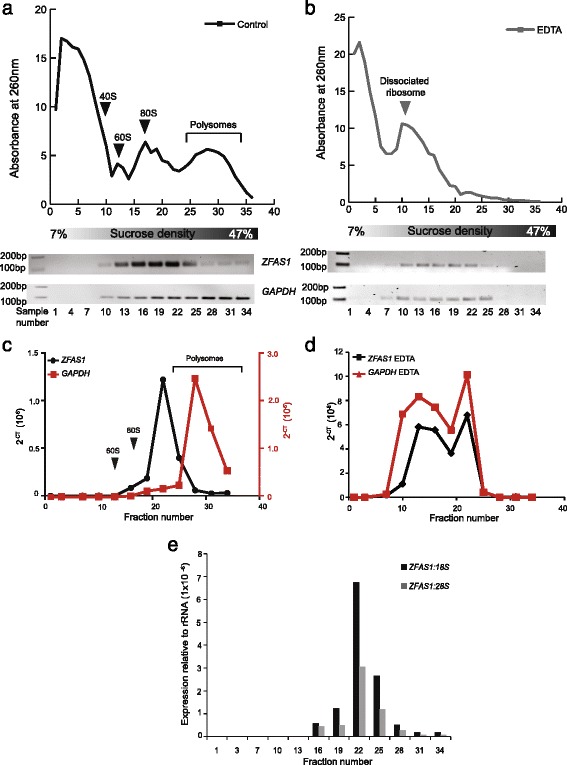
*ZFAS1* is associated with actively translating ribosomes. **a** Polysome distribution of MDA-MB-468 cell lysates as separated on a 7–47% sucrose gradient. Absorbance at 260 nm is shown on the Y axis. Fractions from the top of the gradient to the bottom are shown from left to right on the X axis. Fractions were collected in 36 equal volumes, of which every third was used for RNA extraction, and cDNA synthesised for PCR. The presence of *ZFAS1* expression was assessed using primers described in Fig. 1, with *GAPDH* acting as a positive control. Both species were enriched in the bottom fractions of the gradient, corresponding to polysomes. **b** Polysome distribution of MDA-MB-468 cell lysate separated on a 7–47% sucrose gradient containing EDTA instead of MgCl_2_. Loss of the polysome peak is observed, together with a leftward shift of the ribosome subunits. RT-PCR analysis (lower panels) showed that *ZFAS1* and *GAPDH* are no longer enriched in the lower peaks, and show concomitant shifts to the upper fractions. **c** and **d** Quantitative expression of *ZFAS1* and *GAPDH* measured by qPCR relative to *18S* and *28S* rRNAs prepared with and without the addition of EDTA (black graph for *ZFAS1,* red graph for *GAPDH*). *ZFAS1* and *GAPDH* are no longer enriched in the lower fractions corresponding to polysomes following polysome disruption by EDTA. Arrows indicate where ribosomal features are observed on profiles in relation to fraction number. **e** Ratio of *ZFAS1* to *18S* and *28S* rRNA in fractions from MDA-MB-468 polysome gradients. qPCR was performed using samples from polysome separation and expression of *ZFAS1* relative to *18S* and *28S* was calculated. *ZFAS1* was detected in fractions 16–34 and showed greatest abundance in fraction 22, corresponding to light polysomes. The ratio of *ZFAS1: 18S* is also greater than that of *ZFAS1:28S* for all fractions

Representative fractions from the gradient were assayed for the presence of *ZFAS1* molecules by RT-PCR. *ZFAS1* was found in the bottom half of the gradient, and its distribution matched regions of the A260 nm profile corresponding to the position of 80S ribosomal subunits and light polysomes (Fig. 3a). *GAPDH*, a constitutively translated mRNA, was used as a positive control for association with polysomes.

To distinguish *ZFAS1* interactions with ribosomal components rather than with other non-ribosomal RNA-protein complexes, the sedimentation profile of *ZFAS1* was analysed by RT-PCR after sucrose-density centrifugation in the presence of 15 mM EDTA. The sequestration of Mg^2+^ by EDTA leads to dissociation of ribosomes from mRNAs without disrupting non-ribosomal RNA-protein complexes [20]. Figure 3b shows that EDTA treatment disrupted the ribosome profile, leading to the loss of polysomes and a leftwards shift of the A260-absorbing species, indicating that ribosomes and free mRNAs had dissociated from each other. It also leads to the loss of the mRNA *GAPDH* and of the lncRNA *ZFAS1* from the lower polysome fractions and a concomitant shift of their distribution to the upper fractions of the gradient. These results indicate that the loss of *ZFAS1* from the lower fractions is due to its dissociation from ribosomes, and not from non-ribosomal protein and/or RNA complexes (Fig. 3b), suggesting that *ZFAS1* is a ribosome-bound lncRNA.

Quantitative PCR (qPCR) analysis of these polysomal fractions (Fig. 3c and d) has further highlighted the shift of both *ZFAS1* and *GAPDH* to upper fractions of the gradient in the presence of EDTA as compared to control sucrose gradients. Further analysis of these polysomal fractions has shown that for each *ZFAS1* transcript, there are approximately 500–20,000 transcripts of *18S* rRNA per *ZFAS1*, and 1000–50,000 transcripts of *28S* rRNA depending on the fraction (Fig. 3e). The data suggest that *ZFAS1* is associated with only a small fraction of the ribosomes, and that it is enriched in fractions containing *18S* RNA relative to those containing *28S* rRNA.

### *ZFAS1* binds to the small ribosomal subunit

**Fig. 4:**
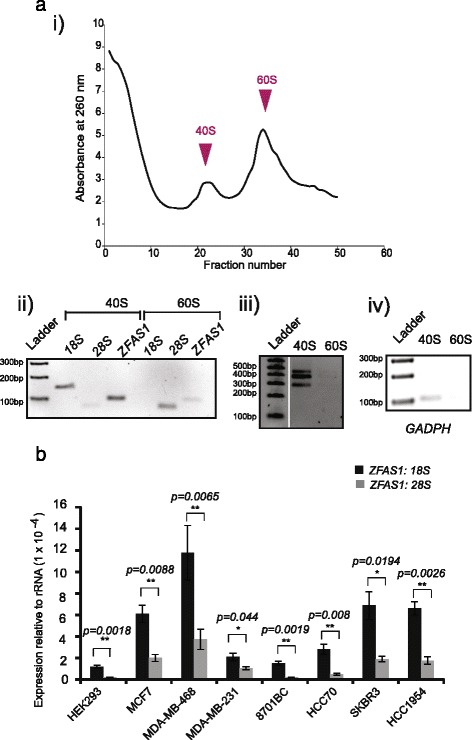
*ZFAS1* is predominantly associated with the small ribosomal subunit. **a** (i) Ribosomal subunit profile of MDA-MB-468 cells. The Y and X axes represent the A260 and fraction numbers respectively. Sample 1 is from the top of the gradient while 50 is the bottom fraction. (ii) *ZFAS1* RT-PCR products using primers as described in Fig. 1 from each peak, fractions 22 and 34. *18S* and *28S* rRNA are used to indicate the small and large subunit distribution in the gradient. (iii) *ZFAS1* RT-PCR products using primers E1F3-E5R1 from each peak, showing that *ZFAS1* is expressed with the 40S subunit in an isoform-independent manner. (iv) *GAPDH* RT-PCR products from each peak, used as a positive control. **b** Ratio of *ZFAS1* to *18S* and *28S* rRNA in different cell lines. qPCR was performed on extracts from various cell lines and expression of *ZFAS1* relative to *18S* and *28S* rRNA was calculated. Error bars represent SEM of 3 biological replicates; p values were calculated using the Mann–Whitney test

To elucidate the role of ribosome-associated *ZFAS1* in regulating ribosome function*,* we sought to identify the subunit with which *ZFAS1* was associated. Crude ribosome pellets from MDA-MB-468 cells were incubated with 0.5 M KCl and 1.5 mM MgCl_2_ buffer and separated in 15–30% sucrose gradients [21]. Fractions from the peaks of the ribosome gradient profile corresponding to 40S and 60S subunits (Fig. 4aai) were isolated and extracted RNA was used for cDNA synthesis and analysis by RT-PCR. The *18S* and *28S* rRNA molecules were used as controls to confirm the identity of the ribosomal subunit that was present in particular fractions and extracted RNA was used for cDNA synthesis and further analysis by PCR. We confirmed that *ZFAS1* is predominantly associated with the small ribosomal subunit (Fig. 4aii), in an isoform-independent manner (Fig. 4a(iii)) similar to *GAPDH* mRNA (Fig. 4(aiv)), and thus is unlikely to play a role in elongation or termination of protein translation which engages the large ribosomal subunit.

Further analysis in different cell lines of *ZFAS1* abundance relative to that of *18S* and *28S* rRNA corrected for dilution factor showed that the ratio of *ZFAS1*:*18S* is greater than that of *ZFAS1*:*28*S (Fig. 4b) with approximately 1500–8000 and 2500–50,000 *18S* and *28S* rRNA molecules to each *ZFAS1* molecule, respectively*.*

### *ZFAS1* expression is correlated with expression of genes encoding proteins involved in ribosome biogenesis

*Zfas1* is differentially expressed during successive stages of mammary gland development [15]. We revisited our original microarray data of mouse mammary gland transcriptomes [15] and found that the expression of several ribosomal protein genes was correlated with *Zfas1* expression during mammary gland development (Table 1). We selected these transcripts for further analysis of human expression data derived from TCGA dataset as described above. The expression of *ZFAS1* was not significantly different in breast cancer patients as compared to healthy controls (*P* = 0.4941), which was the case also with *RPS3* (*P* = 0.14) (Additional file 3: Figure S3A and B).

**Table 1:** Genes encoding ribosomal proteins, expressed differentially during successive stages of mouse mammary gland development and concordantly with *Zfas1* (microarray data)

Target uniGene symbol	Target uniGene name	Log2 fold change (lactating/pregnant)
*Zfas1*	AK005231,BC042795	−5.106
*Rpl22*	Ribosomal protein L22	−6.18
*Rps24*	Ribosomal protein S24	−6.007
*Rps3*	Ribosomal protein S3	−5.665
*Rps21*	Ribosomal protein S21	−5.435
*Rpl28*	Ribosomal protein L28	−4.577

The other ribosomal protein gene transcripts identified in mouse mammary gland to correlate [15] with *ZFAS1,* i.e. *RPS21*, *RPS24*, *RPL22* and *RPL28*, showed significant expression differences between normal and tumour samples (*P < 0.05, **P < 0.01, ***P < 0.001, ****P < 0.0001; Additional file 3: Figure S3D, F, H and J). However, the actual difference in expression between cancer and normal tissue is negligible and the significance reflects the large sample size. The expression of these ribosomal protein genes was strongly and positively correlated with *ZFAS1* expression in normal human tissues (Table 2, Additional file 3: Figure S3) (Spearman correlation, *r* = 0.68-0.85, *p* > 0.001). The expression of these ribosomal protein genes was also correlated with that of *ZFAS1* in breast cancer samples, showing moderate positive correlations (*r* = 0.41–0.60). To confirm that this was not a random phenomenon, genes were randomly selected and possible correlations with *ZFAS1* investigated (Additional file 4: Table S1). These genes exhibited either weak or no correlation with *ZFAS1* indicating that *ZFAS1* is specifically correlated with genes encoding cytoplasmic ribosomal proteins.

**Table 2:** Correlation of *ZFAS1* expression with that of ribosomal protein genes in human (i) non-tumour and (ii) breast cancer samples (TCGA data)

	ZFAS1 vs. RPS3	ZFAS1 vs. RPS21	ZFAS1 vs. RPS24	ZFAS1 vs. RPL11	ZFAS1 vs. RPL22	ZFAS1 vs. RPL28	ZFAS1 vs. MRPL16
i) Normal Tissue							
Pearson r	0.7495	0.7498	0.8053	0.8	0.849	0.6846	−0.1178
*P* value							
*P* (two-tailed)	<0.0001	<0.0001	<0.0001	<0.0001	<0.0001	<0.0001	0.214
*P* value summary	****	****	****	****	****	****	ns
Significant? (alpha = 0.05)	Yes	Yes	Yes	Yes	Yes	Yes	No
Number of XY Pairs	113	113	113	113	113	113	113
ii) Breast tumour tissue							
Pearson r	0.4639	0.6017	0.5429	0.5654	0.4913	0.4988	0.2074
*P* value							
*P* (two-tailed)	<0.0001	<0.0001	<0.0001	<0.0001	<0.0001	<0.0001	<0.0001
*P* value summary	****	****	****	****	****	****	****
Significant? (alpha = 0.05)	Yes	Yes	Yes	Yes	Yes	Yes	Yes
Number of XY Pairs	1049	1049	1049	1049	1049	1049	1049

### *ZFAS1* increases during ribosome biogenesis

**Fig. 5:**
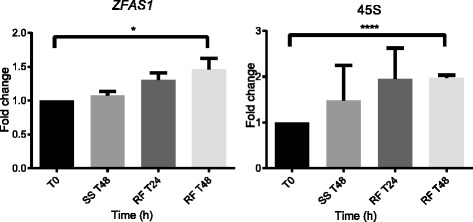
*ZFAS1* is induced concurrently with ribosome biogenesis. MDA-MB-468 cells were maintained for 48 h in medium containing low serum (SS), and then refed (RF) for 24 and 48 h in medium containing serum at 10 %. qPCR was then performed to measure the expression of *ZFAS1* and *45S* rRNA at different time points. Fold change relative to time 0 is shown on the Y axis, and treatment time (h) shown on the X axis. Error bars are SEM of three biological replicates, p values were calculated using Student’s t test

To investigate whether *ZFAS1* expression changes in parallel with transcripts of ribosomal proteins, we induced ribosome biogenesis in MDA-MB-468 cells by starving them for 48 h in medium containing 0.5% serum, followed by refeeding with medium containing 10% serum. *45S*, the pre-rRNA transcript, was used as a marker of ribosome biogenesis. During starvation, expression of *ZFAS1* and *45S* remained the same as in untreated cells. At 48 h after refeeding with serum-supplemented medium, the abundance of *ZFAS1* increased by 46% (*p* = 0.048), and of *45S* increased by 97% (*p* < 0.0001) (Fig. 5).

### *ZFAS1* knockdown: a subtle phenotype with a ribosomal connection

**Fig. 6:**
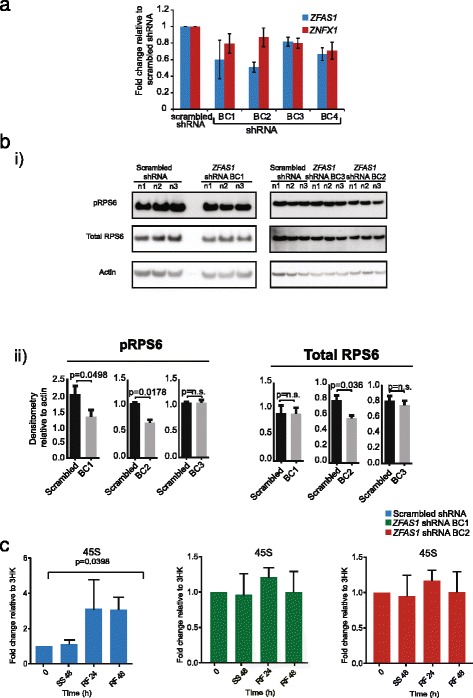
Effect of *ZFAS1* knockdown on cell phenotype. **a** Four different shRNA constructs (BC1-4) designed to target *ZFAS1* were transfected into MDA-MB-468 cells. The efficiency of shRNA knockdown was analysed by qPCR in relation to a scrambled shRNA control. *ZFAS1* transcripts were knocked down to the greatest extent by shRNA BC1 and BC2, these were used for further analysis. **b** (i) Western blot analysis of phosphorylated RPS6 and total RPS6 in *ZFAS1* knockdown and scrambled control cells in three biological replicates. Actin was used as an internal control. (ii) Semiquantitatve analysis of western blot results using densitometry comparison to actin. shRNA BC1–BC3 transfected cells and scrambled shRNA were used. Error bars are SEM of three biological replicates, p values were calculated using Student’s t-test. **c** Cells transfected with scrambled shRNA and *ZFAS1*-shRNA cells were maintained for 48 h in medium containing low serum (SS), and then refed (RF) for 48 h in medium containing serum at 10 %. qPCR was then performed to measure the expression of *45S* rRNA at different time points. Expression is relative to three housekeeping genes (3HK). Error bars are SEM of three biological replicates, p values were calculated using Student’s t test

The MDA-MB-468 cell line was selected for knock down of *ZFAS1* by shRNA as this cell line has moderately high expression of the gene. Four different shRNAs targeting *ZFAS1* as well as a control scrambled shRNA were used. The level of downregulation of *ZFAS1* following shRNA transfection was determined by qPCR using the scrambled shRNA to normalize the expression level (Fig. 6a). The result confirmed that the two shRNAs that achieved the greatest knockdown effects were *ZFAS1* shRNA BC1 and BC2, which achieved reductions of *ZFAS1* to 60 and 50% respectively. These shRNA transfected cell lines were chosen for further analysis.

Previous work in mouse cell lines has suggested that *ZFAS1* acts as a regulator of proliferation [15]. Using cells transfected with *ZFAS1* shRNA BC2 as described above, we measured cell proliferation over 7 days using the SRB assay (Additional file 5: Figure S4A). No significant differences were observed between cells transfected with scrambled control and *ZFAS1* shRNA BC2. Transfection with the scrambled control and *ZFAS1* shRNA BC2 also did not result in significant differences in cell size (Additional file 5: Figure S4B) or global nascent protein synthesis (Additional file 5: Figure S4C).

As *ZFAS1* was found associated with the small ribosomal subunit we postulated that *ZFAS1* may regulate ribosome activity through interactions with components of the 40S subunit. We chose to examine ribosomal protein S6 (RPS6), one of the major proteins on the small ribosomal subunit. It undergoes inducible phosphorylation mediated through TORC1 and promotes protein synthesis [22]. To examine whether *ZFAS1* affects the phosphorylation state of RPS6 we performed Western blot analysis by using total protein from cells containing *ZFAS1* shRNA BC1 and BC2 and scrambled controls shRNA, investigating the relative abundance of phosphorylated RPS6 (the functionally active form) and total RPS6 (Fig. 6bi). Knockdown of *ZFAS1* decreased the phosphorylation level of RPS6 in *ZFAS1* BC1 and BC2 shRNA transfected cells by 36 and 35% respectively (Fig. 6bii). Total RPS6 also decreased in *ZFAS1* BC2 shRNA transfected cells (28%), although to a lesser extent than phospho-RPS6. When compared to BC3, which did not decrease *ZFAS1*, there was no change in either phospho-RPS6 or total RPS6 protein abundance (Fig. 6b). The decrease in abundance and phosphorylation state of RPS6 supports the hypothesis of functional interactions between *ZFAS1* and the 40S subunit.

As *ZFAS1* was induced during ribosome biogenesis, we hypothesised that *ZFAS1* may play a role in ribosome induction. To determine whether induction of ribosome biogenesis was affected by *ZFAS1*, qPCR was performed on cells transfected with *ZFAS1* shRNA BC1, BC2 and scrambled controls after serum starvation and subsequent refeeding with normal medium (Fig. 6c). The abundance of *45S* increased 3-fold (*p* = 0.0398) in scrambled controls 48 h after the reintroduction of normal medium. This response is similar to that shown by non-transfected MDA-MB-468 cells in an earlier experiment (Fig. 5). *ZFAS1* knockdown cells displayed no significant change in *45S* abundance after serum starvation or refeeding, a finding that supports the hypothesis of its role in ribosome production.

### *ZFAS1* (variant 4) has a 5’TOP sequence and may resist NMD

Five different isoforms of *ZFAS1* have been reported (Fig. 2a and d), of which the sequence of the 5’ end is predicted to be repeats of pyrimidines, indicative of the 5’TOP structural motif. 5’-RACE (rapid amplification of cDNA ends) was performed to confirm sequences indicated by the current genomic assembly. Due to the GC-rich nature of the 5’-end of exon 1, only exon 1 of variant 4 was identified (Additional file 6: Figure S5A). This exon aligned to RefSeq Accession Number NR_003604, March 2006 NCBI36/hg18 with a variable number of thymidine residues at the 5’ end found in different transcripts (Additional file 6: Figure S5A).

Tract of pyrimidines (TOP) at the 5’ end is characteristic of a class of RNA targeted to the ribosome. 5’TOP mRNAs encode ribosomal proteins and elongation factors, while other previously studied 5’TOP lncRNAs include transcripts containing snoRNAs in intronic sequences. Upon inhibition of protein synthesis, these transcripts accumulate as they are no longer able to enter the ribosome wherein they would be degraded by the nonsense-mediated decay (NMD) pathway [23]. As *ZFAS1* is host to three snoRNAs as well as containing a 5’TOP, we hypothesised that *ZFAS1* might be regulated by a similar pathway.

To inhibit ribosome function, cell growth was arrested by serum starvation. As previously shown, lncRNA *GAS5* is upregulated during growth arrest [23, 24]. Total RNA from multiple cell lines was tested to determine the expression of *ZFAS1* and *GAS5,* but only HEK293 and MDA-MB-231 showed significant upregulation of *GAS5*, with HEK293 cells showing greater accumulation (15 fold increase) than MDA-MB-231 cells (3 fold increase) at 72 and 48 h respectively. These cells, as well as 8701BC cells, also showed a significant increase in *ZFAS1* (Additional file 6: Figure S5B). Thus, our results suggest that the accumulation of *ZFAS1* and *GAS5* during serum starvation is cell line-specific.

To further investigate the possible role of *ZFAS1* in ribosome function, we treated breast cancer cells with the translation elongation inhibitors puromycin and cycloheximide. Puromycin induced a significant increase of *ZFAS1* abundance in MDA-MB-231, HeLa and MDA-MB-468 cells (Additional file 7: Figure S6), but no significant differences in the abundance of *ZFAS1* following treatment with cycloheximide were detected (Additional file 8: Figure S7). These results suggest that inhibiting ribosomes at specific sites in certain cell lines induces the accumulation of *ZFAS1*.

## Discussion

*Zfas1* was discovered as a lncRNA that is differentially expressed during mouse mammary gland development with the highest level detected during pregnancy [15]. Many antisense lncRNAs regulate the associated protein coding genes in *cis*. The lack of correlation between *ZFAS1* and *ZNFX1* expression indicates that there is no apparent *cis* regulation between them*,* and provides a basis for seeking alternative *ZFAS1* activities. In this study, we have shown that *ZFAS1* is expressed in all cell lines tested, although the abundance varies between cell lines. *ZFAS1* expression is not correlated with that of its protein coding counterpart, *ZNFX1* (Fig. 1b). Analysis of TCGA data indicated that *ZFAS1* expression was not reduced in breast cancers in general (Additional file 2: Figure S2A i), but suggested that particular subtypes (basal, HER2-positive) show reduced expression (Additional file 2: Figure S2A ii, iii).

Studies in hepatocellular carcinoma and colorectal cancer have found that *ZFAS1* is more highly expressed in these cancers as compared to normal tissues [25, 26], and that higher expression of *ZFAS1* is associated with tumour metastasis and poor patient prognosis. It has been hypothesised that *ZFAS1* acts as a miRNA sponge [25, 26]. However, we found no correlation between *ZFAS1* and previously described miRNA target genes [25, 26] in breast cancer according to TCGA data (analysis not shown).

In our current studies, *ZFAS1* was localised to both the cytoplasm and nucleus in a non-isoform specific manner (Fig. 2c and d), as with our previous study of mouse mammary epithelial cells [15], in which only one isoform was detected. Whether different isoforms in human have different functions remains to be elucidated.

Significant findings arising from this study are that *ZFAS1* isoforms are associated with ribosomes (Fig. 3) and are bound to the small 40S subunit (Fig. 4). Many lncRNAs appear to be ribosome-associated [9, 10]. We found that cytoplasmic *ZFAS1* is associated with actively translating ribosomes, with light polysomes showing the greatest concentration of *ZFAS1.* This suggests that *ZFAS1* is associated with only a small proportion of ribosomes at any given time. Ribosomes exhibit heterogeneous composition, which is thought to evince diverse functionality [27]. The widespread association of lncRNAs with ribosomes contributes to this heterogeneity and may reflect possible mechanisms for regulating ribosome function.

Microarray data from mouse mammary glands at different stages of development have shown that *ZFAS1* and genes encoding several ribosomal proteins show similar changes in expression level during pregnancy and lactation (Table 1, Additional file 3: Figure S3). These transcripts for ribosomal proteins, like *ZFAS1,* do not exhibit appreciable differences in expression between human breast cancer and normal breast tissue samples. They do, however, exhibit stronger correlations with *ZFAS1* in normal tissue compared to breast cancer tissue. This may be a reflection of deregulated ribosome function and synthesis that occurs in neoplastic cells [28]. Interestingly, the respective ribosomal proteins have been found to participate in ribosome synthesis as regulators of ribosomal structure [29, 32, 33] and in formation of mature rRNA [30, 31]. Induction of ribosome biogenesis, confirmed by the increased expression of *45S*, also induced expression of *ZFAS1* (Fig. 5). These findings suggest that *ZFAS1* may be involved in early stages of ribosome induction or production.

*ZFAS1* is host to three C/D box snoRNAs which target rRNA for post-transcriptional modification. snoRNAs are often located within the introns of protein-coding genes, many of which have functions in ribosome biogenesis and/or translation [34]. Additionally, at least one of the five *ZFAS1* isoforms contains a 5’TOP motif. 5’TOP function is manifested by selective unloading of mRNAs from polysomes during repression of the cell cycle, or recruitment to polysomes during cell proliferation or refeeding of starved cells [35]. It is possible that lncRNAs with similar motifs also engage in regulatory activities with ribosomes [22].

The lncRNA *GAS5*, like *ZFAS1*, is a member of the 5’TOP gene family, and hosts several snoRNAs. The abundance of *GAS5* transcripts increases during growth arrest and it accumulates with submonosomal fractions [23, 36] due to inhibition of NMD. However, the abundance of *GAS5* increased only in certain cell lines during serum starvation, suggesting that its regulation by NMD is cell line-specific. *ZFAS1* also accumulated in certain cell lines during serum starvation (MDA-MB-231, 8701BC, HEK293) and with puromycin treatment (MDA-MB-468, HeLa, MDA-MB-231), but not with cycloheximide treatment (Additional file 6: Figure S5; Additional file 7: Figure S6 and Additional file 8: Figure S7). Serum starvation causes global ribosomal inhibition, whereas cycloheximide and puromycin target the ribosome at different sites inhibiting elongation of polypeptide synthesis [37, 38], and premature chain release and subsequent dissociation of ribosomes [39] respectively. Such alternative mechanisms may explain why these drugs have different effects on *ZFAS1* abundance, as the dissociation of ribosomal subunits may be required to release *ZFAS1*. Our data suggest that the *GAS5* and *ZFAS1* transcripts function differently in cells despite their similarity in structural motifs.

We sought to identify the possible functions of *ZFAS1* through shRNA knockdown. We were able to achieve 40 and 50% knockdown using different shRNAs (BC1 and BC2), and observed that upon reduced expression of *ZFAS1*, phosphorylation of RPS6 decreased (Fig. 6b). RPS6 is a component of the 40S small subunit, and undergoes phosphorylation induced by anabolic signals. The molecular mechanisms underlying the diverse effects of RPS6 phosphorylation on cellular and organismal physiology are still poorly understood [22].

RPS6 is thought to regulate translation, proliferation and cell size, but we did not observe appreciable differences in *ZFAS1* knockdown cells (Additional file 5: Figure S4). However, S6 kinases, responsible for RPS6 phosphorylation, have been implicated as regulators of ribosome biogenesis factors [39]. RPS6 also has distinct functions in the cytoplasm and nucleus. In the cytoplasm, phosphorylation of RPS6 promotes translation of specific transcripts [40], whereas in the nucleus it binds to the pre-rRNA after transcription as part of the small subunit processome for generation of mature 18S [41, 42]. Knockdown of *ZFAS1* may interfere with this ribosome biogenesis programme reflected in reduced phosphorylation and abundance of RPS6.

Knockdown of *ZFAS1* abolishes increases in 45S abundance after ribosome induction through serum refeeding after prior starvation compared to scrambled control siRNAs (Fig. 6c). This supports a role of *ZFAS1* in ribosome biogenesis and may suggest that *ZFAS1* functions to promote 45S transcription or processing. However, serum starvation affects a plethora of responses, and *ZFAS1* may modulate several divergent pathways.

We have shown that *ZFAS1* binds to ribosomes and in particular, the small 40S subunit. We propose a novel function for this lncRNA, in which it is translocated to the cytoplasm while still associated with the small ribosomal subunit. We suggest that *ZFAS1* may not regulate translation directly, but instead regulate ribosome production and assembly, which adds another layer of complexity to ribosome regulation and function.

The description of ribosome processing and synthesis in humans has lagged far behind that of budding yeast, due to the questionable assumption that these processes are phylogenetically conserved. Studies in yeast experimental systems may have limited applicability to mammalian systems as ribosome biogenesis trends towards increased evolutionary complexity [43].

A large number of lncRNAs associate with ribosomes and may act as possible ribosomal regulators [9–11]. Additionally, multiple ribosomal factors often perform redundant tasks, so investigating the function of a specific lncRNA by knockdown may not exhibit an appreciable effect as it may be compensated by similarly redundant lncRNAs and will require complementary approaches.

## Conclusions

We propose a novel role for lncRNAs in which they associate with ribosomes and regulate their function. The majority of lncRNAs exhibit low stoichiometry and the association of each lncRNA with highly abundant ribosomes may confer fine tuning and selectivity upon ribosome function or synthesis. Our lncRNA of interest*, ZFAS1* is induced upon ribosome biogenesis, suggesting a role in synthesis or assembly of ribosomes. *ZFAS1* knockdown decreases RPS6 phosphorylation and ribosome biogenesis induction, suggesting that *ZFAS1*, in its association with the 40S subunit, may not regulate translation directly and instead may be involved in ribosome production and assembly.

## Methods

### Cell culture

Cells were sourced and cultured as described previously [44].

### RNA extraction, reverse transcription, qPCR and PCR

RNA extraction has been described previously [15]. Briefly, total RNA from cultured cells was purified using Trizol (Life Technologies). To remove genomic DNA, total RNA was treated with DNAse I for 30 min at 37 °C followed by incubation at 75 °C for 10 min to deactivate DNAse I. To assess the quality and yield of RNA, absorbance at 260, 280 and 230 nm was measured with a Nanodrop 1000 spectrophotometer. The ratios of optical density at 260/230 and 260/280 nm were ≥ 1.8 in all cases.

Random hexamers or oligo dT were used to reverse transcribe 1 μg of RNA with M-MLV Reverse Transcriptase (Sigma) according to the manufacturer’s instructions. Oligo-dT was used for the preparation of cDNA used for analysis of *ZFAS1* and *ZNFX1* expression in breast cancer cell lines, whereas random hexamers were used for the remaining experiments.

For non-quantitative analysis, cDNA was diluted 1:20 and PCR-amplified for 30 cycles in a PCR thermocycler (95 °C for 5 min, followed by 30 cycles of 95 °C for 30 s, 60 °C for 30 s, 72 °C for 1 min/kb, followed by 72 °C for 5 min). For PCR of exon 1, Kapa Hifi Taq was used according to the manufacturer’s instructions with a touch down procedure [45] (98 °C for 5 min, followed by 10 cycles of 98 °C for 20 s, 75 °C for 15 s and 72 °C for 30 s in which annealing temperature was decreased by 1 °C each cycle). After this, PCR products were amplified by 30 cycles of 98 °C for 20 s, 65 °C for 15 s and 72 °C for 30 s). Products were visualised on a 2% agarose gel stained with ethidium bromide. The list of primer sequences is provided in Additional file 9: Table S2, and the sizes of products were estimated by running a 1 kb ladder alongside the PCR products.

For quantitative PCR, a 1:20 dilution of cDNA was used to measure the abundance of all transcripts studied here except for the *18S* and *28S* rRNAs. In the latter cases, 1:1000 dilutions were used. Reactions contained SYBR Green PCR master mix (Applied Biosystems), and primers were diluted to 8 μM. Cycling conditions are detailed in ref [15]. For all qPCR data, experiments were performed three times as biological replicates.

### 5′ Rapid Amplification of cDNA Ends (RACE)

RNA derived from MDA-MB-468 was converted into cDNA as described above and tailed with poly G using terminal transferase according to the manufacturer’s description (New England Bio labs, Cat. No. M0315S). PCR was performed as described above using primers AN polyC + E2R2 for 10 cycles with an annealing temperature of 65 °C, after which 1 μL of the PCR reaction was used as the template for a subsequent PCR using AN F and E2R1 primers for 30 cycles at an annealing temperature of 65 °C. PCR samples were purified and ligated into pGEMT Easy Vector and cloned into *E.coli* cells. Colonies positive for the insert were selected and used as a template for PCR with M13 Forward and M13 Reverse primers. Those PCR samples which demonstrated the presence of the insert were then selected for sequencing.

### Subcellular fractionation

Three 175 cm^2^ flasks of MDA-MB-468 or MDA-MB-231 cells were grown to 80% confluency then trypsinised, and the cells pelleted at 110 g for 5 min. The cell pellet was washed with ice-cold phosphate-buffered saline (PBS), and pelleted again. The pellet was then resuspended in 5 mL ice-cold fractionation hypotonic lysis buffer (10 mM HEPES pH 8.0, 1.5 mM MgCl_2_, 10 mM KCl, 0.5 mM DTT, 1x protease inhibitor (Sigma Aldrich cat no. P8340) and incubated on ice for 5 min. Cells were then lysed with 20 strokes of a Dounce homogeniser using a tight pestle. The lysate was centrifuged at 228 x g for 5 min at 4 °C.

The supernatant was retained as the cytoplasmic fraction. Total cytoplasmic RNA was extracted from this fraction using Trizol. The pellet was resuspended in 3 mL of 0.25 M sucrose containing 10 mM MgCl_2_, and the extract layered over 3 mL 0.88 M sucrose, 0.5 mM MgCl_2_ followed by centrifugation at 2800 x g for 10 min at 4 °C. The supernatant was discarded and the pellet was designated the nuclear fraction, which was then suspended in 1 mL Trizol for RNA isolation. Experiments were performed twice for biological replicates.

### Polysome analysis

Polysome fractionation was performed with minor modifications as described in ref [46]. In detail, twelve 175 cm^2^ flasks of MDA-MB-468 breast cancer cells were grown to 80% confluency then incubated with cycloheximide (100 μg/mL) in PBS for 15 min at 37 °C before harvesting by trypsinisation and centrifugation at 110 g for 5 min. The cell pellet was resuspended in 2 mL polysome lysis buffer (20 mM HEPES pH 7.4, 15 mM MgCl_2_, 200 mM KCl, 1% Triton X-100 (v/v), 100 μg/mL cycloheximide, 2 mM DTT, 1 mg/mL heparin) and lysed with 20 strokes of a dounce homogeniser using a tight pestle. Lysate was cleared by centrifugation at 14,000 x g for 5 min at 4 °C. Cleared cell lysate was layered upon 7–47% (w/v) sucrose gradients, prepared as described in ref [47] in 50 mM NH_4_Cl, 50 mM Tris–HCl pH 7.0 and 12 mM MgCl_2_ in polyallomer tubes (Beckman) and loaded into a SureSpin™ 630 rotor and centrifuged in a Sorvall Ultracentrifuge at 100,000 x g for 4 h at 4 °C. After centrifugation, gradients were fractionated by securing the ultracentrifuge tube in a clamp stand and piercing the bottom of the tube with a 21G needle. Drops of the sucrose gradients flowed at a consistent rate (approximately 300 μL per fraction) into the wells of a 96 well plate, and absorbance measured at 260 nm using a Nanodrop 1000 spectrophotometer. RNA from every third fraction was purified using Trizol according to the manufacturer’s instructions and used as a template for cDNA synthesis.

For polysome release experiments, the above protocol was followed, except that MgCl_2_ was not present in the lysis buffer or the sucrose gradient, and was replaced with 15 mM EDTA. Both experiments were performed twice to provide biological replicates.

### Ribosome subunit separation

Eight 175 cm^2^ flasks of MDA-MB-468 cells were grown to 80% confluency and the cells collected by trypsinisation followed by centrifugation. The cell pellet was resuspended in 2 mL lysis buffer (20 mM Tris–HCl pH 7.5, 5 mM MgCl_2_, 100 mM KCl, 0.5% NP40, 10 mM 2-mercaptoethanol, 1x protease inhibitor and 100 U/mL RNAse inhibitor (Roche, cat no 03335 399001). Cells were lysed with 20 strokes of a Dounce homogeniser and cell lysate was cleared by centrifugation at 12000 x g for 20 min. One-tenth of the volume of 10% w/v sodium deoxycholate was then added to release ribosomes from microsomal membranes. Cleared cell lysate was layered on a 50 mL 1 M sucrose cushion in 50 mM Tris–HCl pH 7.5, 5 mM MgCl_2_, 100 mM KCl, 10 mM 2-mercaptoethanol and centrifuged for 16 h at 100,000 x g in a JA-30.50Ti ultracentrifuge rotor (Beckman Coulter). Supernatant was removed, and the ribosome pellet was resuspended in 1 mL ribosome buffer (5 mM Tris HCl pH 7.5, 1.5 mM MgCl_2_, 50 mM KCl, 10 mM 2-mercaptoethanol). KCl and puromycin were added to the ribosome suspension to final concentrations of 0.5 M and 1 mM respectively, then incubated on ice for 30 min, followed by incubation at 37 °C for 15 min. The ribosome suspension was clarified by centrifugation for 5 min at 10,000 × g.

The cleared ribosome suspension was then layered on a 15–30% sucrose gradient in 5 mM Tris–HCl pH 7.5, 5 mM MgCl_2_, 0.5 M KCl, 10 mM 2-mercaptoethanol, then centrifuged at 30,000 x rpm in a SureSpin™ 630 rotor for 14 h. Samples were collected as detailed in ‘Polysome analysis’.

### Induction of ribosome biogenesis

MDA-MB-468 cells were plated in T25 flasks at 60% confluency. Medium was aspirated, cells washed twice with PBS, and cells cultured with 0.5% FCS-containing medium for 48 h. After 48 h, 10% FCS-containing medium was added for a further 48 h.

### Knockdown of *ZFAS1*

shRNA constructs to knock down *ZFAS1* and control empty plasmid (vector) were purchased from GeneCopoeia. Breast cancer cells (MDA-MB-468) were grown to 80% confluency in 6 well plates and were transfected with 4 μg or 5 μg of DNA using Lipofectamine 3000 in accordance with the manufacturer’s instructions. Transfected cells were selected as described in ref [44].

### Cell proliferation assay

To measure cellular proliferation, the sulforhodamine B colorimetric assay was used, which measures total cellular protein to measure cell density. MDA-MB-468 cells containing constructs for *ZFAS1* shRNA and scrambled shRNA were seeded in 96 well plates at 1500, 3000, 6000 cells per well and processed as described in ref [44] to compare differences in cell growth. Experiments were performed twice for biological replicates.

### Cell size measurement

MDA-MB-468 cells were grown in T25 flasks at 70-80 confluency. Adherent cells were trypsinised resuspend in 1 mL of PBS and diluted 100x in saline. Cells were counted and the size distribution determined using a Coulter Particle Count and Size Analyser (Beckman Coulter model Z2).

### Measurement of nascent protein synthesis

Synthesis of nascent polypeptides was measured using Click-iT® Metabolic Labelling with L-azidohomoalanine (AHA) (ThermoFisher). Cells were plated in 96 well plates as technical duplicates at 30,000 cells per well to achieve 70–80% confluency. Cells were washed with warm PBS, and the medium replaced with methionine free DMEM + 10% FCS and incubated at 37 °C for 1 h to deplete methionine reserves. Cells were further incubated with 40 μM of Click-iT® AHA at 37 °C for 1 h in the dark. Cells were then washed in PBS, fixed with 4% paraformaldehyde in PBS for 15 min, and permeabilised with 0.25% Triton® X-100 for 15 min, after which cells were washed in 3% (w/v) BSA in PBS. Cells were then ready for the detection reaction with alkyne tagged detection molecule. For this, cells were incubated in the dark with 10 mM TBS, 1 mM CuSO_4_, 100 mM sodium ascorbate and 10 μM Alexa Fluor®647 alkyne for 30 min. Nascent protein synthesis was determined by the fluorescence of Alexa 647 using a BD FACS Vantage Cytometer.

### Western blot

Cells were lysed in buffer containing 60 mM Tris–HCl, pH 6.8, 2% (w/v) SDS, and 20% (v/v) glycerol, and protein quantitated by BCA assay. Cell lysates containing 25 μg of protein were separated by SDS-PAGE, and transferred to PVDF membranes (Millipore). Membranes were immunoblotted with antibodies against phospho-S6 ribosomal protein (Ser240/244) (Cell Signalling Cat. No. 2215) (1:1000 for anti-phospho RPS6), total RPS6 (Cell Signalling Cat. No. 2217) and β-actin (1:5000) (Sigma). Protein bound primary antibody was subsequently incubated with respective secondary antibody prior to membrane exposure to SuperSignal West Pico (Thermo Scientific) for β -actin or ECL plus for phospho-RPS6 (Thermo Scientific). Resulting bands were detected using chemiluminescence detection system (Fujifilm Las-3000).

### Statistical analysis

Statistical analysis was performed using GraphPad Prism. Data were analysed using Mann Whitney test, where *p* < 0.05 denotes a statistically significant difference. Pearson correlation test was used for correlation analysis.

### Growth arrest and inhibition of translation

Cells were cultured in media containing 0.5% fetal calf serum, grown to 70-80% confluency and then washed twice with PBS and RNA collected by Trizol extraction at 0, 24, 48, 72 h after medium replacement. For inhibition of translation experiments, cells were treated with 20 μg/mL of cycloheximide or 50 μg/mL of puromycin for 0 to 16 h. Cells were collected and RNA extracted and processed as described above, for cDNA synthesis and qPCR.

### Random selection of genes

Genes were randomly selected from Homo Sapien data set using Random Gene Set Generator from http://www.molbiotools.com/.

## Reviewers’ comments

### Reviewer’s report 1: Christine Vande Velde, Department of Neurosciences, University of Montreal

#### Reviewer summary

This manuscript demonstrates a novel role for a long noncoding RNa (lncRNA) called ZFAS1. The authors establish the validity of the previously made claim that ZFAS1 is, in fact, a lncRNA, ie. there is no peptide produced. The authors present clear evidence that five different isoforms exist and, despite prediction that different isoforms might exhibit different subcellular localization, there is no difference between them with regards to their nucleocytoplasmic distribution. Hansji and colleagues also present good quality data which indicates that, like some other lncRNAs, ZFAS1 is associated with polysomes. Additional data demonstrate that it is the small ribosomal subunit and the association of ZFAS1 to this subunit can be triggered by growth arrest and inhibition of translation (via puromycin treatment). Lastly, it is demonstrated that siRNA-mediated knockdown of ZFAS1 reduces phosphorylation of the ribosomal protein RPS6 and reduces 45S rRNA, indicating that ZFAS1 is important for ribosome biogenesis. Overall, the quality of the individual experiments is high and each is well controlled. The interpretation of the data is accurate, with minimal/no overstatements.

## Reviewer comments

Given that the authors describe five different isoforms, I was surprised to see them largely ignored after Fig. 2. Thus, in Figs. 3, 4, 5 and 6, it appears that only a single isoform has been examined. Moreover, it is unclear which isoform(s) has been followed (ie. Which primer pair was used in these figures?). This information should be explicitly stated.

Authors’ response: *Primer pairs for qPCR have been specified in the Fig.* *1* *a legend [1], and cover 4 of the 5 isoforms, so representing the majority of ZFAS1 isoforms. Primers are restated in Fig.* *3* *legend.*

The same primer set was used for ribosome gradients, as stated in the legend of Fig. 4. To address the issue of particular isoforms being excluded from consideration, we have attached Additional file 10: Figure S8 to demonstrate different isoforms of *ZFAS1* in ribosome gradients using primers that cover 4 of the 5 isoforms (Primer set E1F3-E5R1).

## Reviewer comments

In addition, it is unclear which isoform(s) is targeted by the siRNAs used to demonstrate effect of ZFAS1 on 45S and rpS6. (That only 1 of the 4 siRNAs gave a robust knockdown could indicate that there is differential role for the various isoforms in this process.)

Authors’ response: *All siRNAs targeted exon 5, which is common for all isoforms, as depicted in Additional file* *11* *: Figure S9. The shRNA should therefore target all isoforms. shRNA sequences targeting ZFAS1 are included in the Additional file* *12* *: Table S3.*

## Reviewer comments

Also, for the sake of completeness, the authors may consider evaluating the presence of the different isoforms in the 40S containing fractions.

Authors’ response: *The presence of different isoforms has been evaluated using primer set E1F3-E5R1 as added in Fig.* *4* *a (iii). ZFAS1 isoforms are located with the 40S subunit extract in a non-isoform specific manner.*

## Reviewer comments

The authors have focused on breast cancer cell lines, due to previous data linking ZFAS1 to mammary gland tissue development, and a previous report of decreased ZFAS1 in invasive ductal breast carcinoma in humans. The authors report here that the expression of a number of ribosomal protein genes correlates with ZFAS1 expression during mammary gland development. However, here ZFAS1 was not found to be significantly different in breast cancer patients compared to healthy controls. Despite this, there was a positive correlation between ZFAS1 expression and a number of these ribosomal protein genes. Authors suggest this discrepancy is due to sample size differences.

Authors’ response: *The data reported in the previous paper were derived from microdissected samples from matched normal and IDC tissue. The RNA from stroma was not included in sample preparation. A total number of five samples was used. Expression patterns showed approximately two fold down-regulation with no statistically significant differences in the values. On the other hand, TCGA data are not derived from necessarily matched-normal samples, and the stromal tissue was included in expression analysis. The correlation of the ribosomal protein genes is strong, also due to large sample size (n = 1043 for breast cancer samples, n = 113 for normal breast tissue samples)*.

However, analyses on different breast cancer subtypes, depicted in Additional file 2: Figure S2A(ii), show that *ZFAS1* is expressed more highly in normal tissues compared to basal and HER2 breast cancer subtypes. It is also more highly expressed in ER+ compared to ER- subtypes (Additional file 2: Figure S2A(iii)). As only 5 samples were measured [1], samples having these characteristics may have been disproportionally represented compared to TCGA samples.

The role of *ZFAS1* as a potential tumour suppressor gene may not be apparent in its expression in all samples in TCGA, but may be reflected in patient survival. Additional file 2: Figure S2B displays a Kaplan-Meier plot generated from http://www.oncolnc.org/ of TCGA breast cancer data set. High expressers are those 50% of patients with the highest *ZFAS1* expression, and low expressers are those 50% of patients with the lowest *ZFAS1* expression. High expressers of *ZFAS1* display increased survival up to 6000 days, which could be an indicative of higher expression of *ZFAS1* in normal samples, although the difference is not significant.

In the microarray data presented previously we have observed differential expression of *Zfas1* and ribosomal proteins during normal mammary gland development in mouse. RNA samples derived for microarray analysis were mainly isolated epithelial cells of the developing glands (see methods in [1]). In TCGA data we did not see any differential expression among these genes (Additional file 3: Figure S3), however the differences were oberved in breast cancer sybtypes compared to normal (Additional file 2: Figure S2). Various patterns of expression observed for these proteins in different subtypes compares to normal. In mouse mammary gland we have see similar pattern of expression for and the ribosomal proteins and *Zfas1* which are consistance with some of breast cancer data derived from TCGA. The two systems examinded in these papers are identical in both species therefore some discripency observed in normal mammary gland development and human breast cancer is not unexpected.

## Reviewer comments

**minor comments**

The connection between the breast cancer cell line analysis and the role for ZFAS1 in ribosome biogenesis becomes evident only on page 15, with the comment that “deregulated ribosome function and synthesis occurs in neoplastic cells.” Could be helpful to highlight this earlier so that links between breast cancer work and ribosomal work are evident early on.

Authors’ response: *We have added sentences introducing the role of ribosomes in cancer progression in Introduction section, page 3 lines 50-52, page 5-6 lines 106-115.*

We have introduced our hypothesis implicating ribosome production in the background (page 3, line 50-52). Our serum starvation data also showed the link between *ZFAS1* and ribosome (Figs. 5 and 6f).

## Reviewer comments

Comments that total rpS6 is unchanged by ZFAS1 shRNA is not well supported by the representative image provided (Fig. 6e):

Authors’ response: *Western blot has been repeated. While total RPS6 does show a decrease in knockdown cells compared to scrambled shRNA (28%), it is less significant than the down regulation of phospho RPS6 compared to scrambled shRNA (35%). We have added Additional file* 8 *: Figure S7 that shows the three biological replicates of the experiment.*

## Reviewer comments

Discussion should be shortened and kept close to the data at hand (it starts to ramble on page 17/18). Also, I would suggest that the authors modify the last few sentences describing what they will do/are doing for the next studies of ZFAS1.

These details are not needed and could be presented in a more generalized way. Abstract and Conclusion lacks any information related to the breast cancer angle of the story. Frequently the “.” comes before the references. This should be corrected. (ie. it should be like this [1]. not like this. [1])

Authors’ response: *Discussion has been shortened.*

References have been corrected.

### Reviewer’s report 2: Nicola Aceto, Department of Biomedicine, University of Basel

#### Reviewer’s summary

Hansji et al. present a well-written manuscript in which they study the long noncoding RNA ZFAS1 in breast cancer cells. They suggest that ZFAS1 is co-localized with polysomes, and predominantly associated with the small ribosomal subunit. Further, they propose a mechanism in which ZFAS1 is required for the production of 45S rRNA as well as for RPS6 phosphorylation in breast cancer cells. I feel that this manuscript would benefit from a more substantial description of the rationale for studying ZFAS1, as opposed to any other long noncoding RNA. Further, additional experiments are needed to reinforce some of the findings (summarised below). Discrepancy between TCGA and previous studies in regard to ZFAS1 expression in normal breast cells versus breast cancer cells should also be addressed in greater detail.

## Reviewer comments

(1) Figure 1b: in the text the authors claim that there is “trend of higher expression of ZFAS1 in normal breast cells”, compared to cancer cell lines. Since they only analyze two normal cell lines (one of them having very similar levels of ZFAS1 as compared to cancer cells), they should avoid indicating that there is a trend. Since no statistical significance is reached comparing ZFAS1 expression in normal versus cancer cells, I would suggest them not to overinterpret the data in this case.

Authors’ response: *This has been removed.*

## Reviewer comments

(2) Figure 1b: can the authors exclude (experimentally) that the difference in expression of ZFAS1 and ZNFX1 is due to different primer efficiency? It would be more convincing to test at least two independent primer sets for each gene, as well as to validate the primers in controlled conditions (e.g. in a setting where ZFAS1/ZNFX1 are downregulated and/or overexpressed).

Authors’ response: *Primer efficiency of ZNFX1 reactions is included in Additional file* *13* *: Figure S10A and B. Primer efficiency is the same as for ZFAS1, indicating that differences in expression level are unlikely to be contributed by the rate of amplification.*

A second set of primers to investigate *ZFAS1* and *ZNFX1* expression has also been designed to ensure different expression is not due to primers. Expression of *ZFAS1* and *ZNFX1* has been evaluated in breast epithelial and breast cancer cell lines using these new sets of primers showing nearly identical patterns of expression. Correlations of qPCR results using the original and new primers is also shown in Additional file 13: Figure S10C.

The expression of *ZFAS1* and *ZNFX1* are shown in Fig. 6a when *ZFAS1* is downregulated by shRNA.

## Reviewer comments

Also why is it important to determine the relative expression difference between ZFAS1 and ZNFX1?

Authors’ response: *The function of lncRNA in cis has been shown in many cases (reviewed in [2]); therefore it is important to determine the relative expression of ZFAS1 and ZNFX1 in the same cells. Antisense lncRNAs are known to regulate the expression of their protein coding counterparts (acting in cis) and we wanted to determine if this was the case for ZFAS1 [3]. Discrepancy in expression level and lack of correlation suggest that they are not co-regulated and ZFAS1 does not regulate ZNFX1. Additionally, lncRNAs often show low expression, so the high expression of ZFAS1 is surprising and potentially functionally important in constitutive processes within the cell such as ribosome biogenesis.*

## Reviewer comments

(3) The TCGA analysis showed no difference in ZFAS1 expression between breast cancer and normal breast tissue. How do the authors interpret this discrepancy with previous results (Askarian-Amiri et al., RNA, 2011)? And how do they motivate their interest in elucidating ZFAS1 biology in the context of normal breast vs breast cancer cells? The rationale for studying ZFAS1 needs to be better explained, in light of the TCGA results.

Authors’ response: *See above comments in response to reviewer 1.*

The gene expression in stromal cells in the microenvironment may have masked the level of gene expression of breast cancer cells.

Since developmental genes are often involved in cancer progression, and our observation of significant down-regulation of *Zfas1* from pregnancy to lactation suggested important developmental roles, we believe that pursuing the function of *ZFAS1* in human and investigating its role in cancer would be challenging but worthwhile.

## Reviewer comments

(4) Figure 5: the authors conclude that ZFAS1 increases during ribosome biogenesis. However, induction of ZFAS1 and 45S rRNA could be serum-induced, yet functionally disconnected events. Since the stimulation with 10% serum has an impact on a wide variety of signaling pathways (many of which not directly connected to ribosome biogenesis), the authors should at least discuss the possible limitations of this particular experiment.

Authors’ response: *This has been addressed in the discussion, page 17 (page 18, line 441-442). However, the use of the ZFAS1 shRNA BC2 to suppress ZFAS1 abundance did appear to limit the induction of 45S rRNA following serum refeeding (Fig.* *6* *f) and one interpretation of this effect is that there are mechanistic connections between the ZFAS1 and ribosomal responses.*

## Reviewer comments

(5) Figure 6e: Difference in p-rpS6 is not striking, and would deserve band quantification and at least *n* = 3 to determine whether or not statistically sound. Also, the authors should include a second shRNA (e.g. BC1) to exclude that their results are due to off-target effects of BC2 shRNA sequence.

Authors’ response: *Multiple shRNAs targeting ZFAS1 were used individually as detailed in Fig.* *6* *a, as well as in combination. ZFAS1 shRNA BC2 was selected because it most effectively knocked down ZFAS1 (to 50%), while other shRNAs showed only 19–40% knockdown. Thus we were unable to include a second shRNA.*

We have repeated the western blot analysis in three biological replicate and performed semi-quantification of the results. We have shown a 35% reduction in phospho-RPS6 as shown in Additional file 8: Figure S7. We also included second *ZFAS1* shRNA BC3 to exclude the possiblilty of false positive result.

## Reviewer comments

(6) Figure 6b: here too, the authors should include a second shRNA in their experiment.

Authors’ response: *SRB assay to measure cell proliferation was repeated, using scrambled shRNA, BC2 and a second ZFAS1 shRNA BC3. This is shown below, with no significant difference in cellular proliferation observed (Additional file* *5* *: Figure S4).*

(7) Given that BC2 sequence enables a 50% knockdown of ZFAS1, wouldn’t the authors expect only a partial blockage of 45S rRNA synthesis in their cells upon expression of BC2?

Yes, blockage of 45S synthesis would be lethal. *ZFAS1* down-regulation may decrease the amount of 45S produced, as shown by lack of induction of 45S upon serum refeeding as shown in Fig. 6f.

**minor comments:**

Figure 6: the authors refer to ZFAS1 shRNA in two different ways: in panels C-D-E as “ZFAS1 shRNA”, and in panel F as “BC2 knockdown shRNA”. Authors should instead use “ZFAS1 shRNA” throughout the text.

Authors’ response: *This has been changed.*

### Reviewer report 3: Haruhiko Siomi, Department of Molecular Biology, Keio University

#### Reviewer’s summary

In sum, results shown in the manuscript are not well connected with each other and are preliminary.

## Reviewer comments

Major comments: Recent studies have shown that a large portion of our genome is transcribed to produce a number of long non-coding RNAs (lncRNAs). However, the functionality of only a small number of these lncRNAs has been demonstrated. This manuscript continues the Askarian-Amiri lab’s analysis of Zfas1, a lncRNA that is abundantly expressed in mouse mammary glands. The authors here characterize Zfas1 in human cells. Their analyses reveal that Zfas1 transcripts may be associated with ribosomes in the cytoplasm. The authors show that Zfas1 may also affect both expression levels of several mRNAs encoding ribosomal proteins and phosphorylation levels of rpS6. These results may suggest a model in which Zfas1 might be involved in ribosome biogenesis through a hitherto unknown mechanism. Major comments: 1. The first parts of this paper (Figs. 1 and 2) mostly repeat data already published by the authors (Askarian-Amiri et al., RNA 2011) though, the authors characterize the human homolog of Zfas1 here. The results shown in Figs. 3, 4, 5 and 6 are to a large extent difficult to understand. It is often unclear what the underlying reasoning is. I simply have a hard time to follow the logic of these analyses. It seems as if the authors just wanted to show what they find with Zfas1 with no clear interpretation.

Authors’ response: *Figure* *1* *a is an image derived from the UCSC browser in [1] and current manuscript, though panel B has not been reported previously. We used three cell lines in our previous article [1]. However, to establish the connection between ZFAS1 and ZNFX1 in this manuscript, we have examined their expression in 21 cell lines and the results presented here confirmed that there was no correlation between the expression of these two genes.*

In our previous paper [1] the relative abundance of alternate isoforms of *ZFAS1* in various human tissues and cell lines, based on exon-exon junction spanning deep sequence tags, was indicated in Fig. 5c ([1]). In this paper we confirm the existence of at least 5 isoforms in breast cancer cell lines tested.

Figures 3, 4, 5 and 6

Figure 3. Experiments were performed to isolate *ZFAS1* and the molecules or supramolecular complexes with which it might be associated. For this purpose we used sucrose gradients and identified *ZFAS1* in 80S-light polysome fractions (Fig. 3a). To confirm the association of *ZFAS1* with ribosomes we treated the lysate with EDTA and loaded it on the gradient. To clarify the shift of peaks upon EDTA treatment, we have changed the scale of the Y-axis in the revised manuscript, confirming a clear difference between panels C and D in Fig. 3.

In Fig. 4, we identified the ribosomal subunit associated with *ZFAS1*. Also the ratios of *ZFAS1* to *18S* and *28S* rRNA in different cell lines were calculated.

Figure 5 confirms that upon *45S* induction, *ZFAS1* is also induced in the system as explained in page 12 of the manuscript (refer to the section ‘*ZFAS1* increases during ribosome biogenesis’, page 12 of the manuscript).

Figure 6. We have performed shRNA knockdown in MDA-MB-468 breast cancer cells to examine the phenotype arising from gene manipulation. Results showed that there was no difference in cell proliferation, cell size and *de novo* protein synthesis. However the level of RPS6 phosphorylation is reduced in knockdown cells. Also we have seen same pattern of gene expression for 45S rRNA in cells transfected with scrambled shRNA (Fig. 6f, left panel) as observed for non-transfected cells (Fig. 5) and this pattern was not detected in shRNA transfected cells (Fig. 6f, right panel).

In summary, we have described the expression patterns of *ZFAS1* in different cancer cell lines and its association with ribosomes (page 8-10). We have tested the association of *ZFAS1*with ribosomes following EDTA treatment and ribosomal subunit dissociation (page 10-11). This confirmed *ZFAS1* association with the small subunit. We have shown how *ZFAS1* and *45S* are co-regulated during ribosomal biogenesis (page 11-12). Following that we used shRNA to knockdown *ZFAS1*. Although we did not find any change in growth rate or global *de novo* protein synthesis, we showed that cells with reduced *ZFAS1* content cannot upregulate *45S* as observed for non- transfected cells, or cells transfected with scrambled shRNA. We also showed RPS6 phosphorylation is reduced in knockdown cells (page 12-13).

## Reviewer comments

For example, the authors state in page 9 that “ZFAS1 is associated with actively translating ribosomes (Fig. 3a).” However, the majority of ZFAS1 cosedimented with 80S but not with polysomes.

Authors’ response: *qPCR analysis of ribosomal fractions shows that ZFAS1 is associated with 80S monosomes and (predominantly) light polysomes, with low association with heavy polysomes (Fig.* *3* *c). We have deleted the “suggesting that ZFAS1 is associated with actively translating ribosome” and added light polysomes instead of polysomes in the same sentence.*

## Reviewer comments

In addition, the distribution profiles of Zfas1 on sucrose density gradients with/without EDTA (Fig. 3a and b) appear very similar each other, suggesting that Zfas1 is not associated with ribosomes.

Authors’ response: *To clarify the difference in distribution of ZFAS1 in those gradients we have performed qPCR of ZFAS1 in all samples. Depiction of EDTA-containing gradients with an expanded y-axis scale shows marked re-distribution from the polysomes to the dissociated ribosomes (Fig.* *3* *d).*

## Reviewer comments

Figure 4 shows that ZFAS1 is more concentrated in 40S fractions (fraction 22) than in 60S fractions (fraction 34). However, we aren’t assured that fraction 5 or 45, for example, may contain Zfas1 as much as does fraction 22.

Authors’ response: *The graphs in Additional file* *14* *: Figure S11 show ZFAS1 expression in fractions derived from the gradient. 18S rRNA is distributed mainly near the top half of the gradient, with 28S distributed near the bottom half. Peaks corresponding to the 40S and 60S subunits are highlighted in red. ZFAS1 is mainly expressed in those fractions corresponding with the 18S (Additional file* *14* *: Figure S11).*

## Reviewer comments

2. Although the authors demonstrated that transfection with the control and Zfas1 shRNA did not result in significant differences in cell size or global protein synthesis (Fig. 6), they found that knockdown of Zfas1 decreases the phosphorylation level of rpS6. Phosphorylation of the protein normally promotes protein synthesis. How would they interpret these results? Does overexpression of Zfas1 increase the phosphorylation level of rpS6?

Authors’ response: *Here we tested de novo protein synthesis. The role of RPS6 in protein synthesis has not been fully resolved, with the exact function of its phosphorylated form still a matter of debate [4]. However, RPS6 has been found to act in the nucleus as a regulator of ribosome biogenesis and its reduced phosphorylation following ZFAS1 knockdown may reflect this. The observed change in phospho-RPS6 content may also reflect its nuclear function [4]. ZFAS1 is highly expressed gene, we are not sure overexpression of it can manifest the real effect or would be artifact.*

## Reviewer comments

The authors also found that Zfas1 knockdown cells displayed no significant change in 45S rRNA abundance after serum starvation or refeeding, though the abundance of 45S rRNA increased 3-fold in controls 48 h after the reintroduction of normal media (refeeding). This suggests that Zfas1 may play role in induction of 45S rRNA. The authors shall consider examining processing and maturation of 45S rRNA because Zfas1 is present in the nucleus as well.

Authors’ response: *Ribosome biogenesis may well include processing and maturation of precursor rRNA transcripts. We fully acknowledge the potential importance of these processses and are currently performing more experiments to consider the suggested functions. Also in this manuscript we have mainly focused on cytoplasmic ZFAS1.*

1. Askarian-Amiri ME, Crawford J, French JD, Smart CE, Smith MA, Clark MB, Ru K, Mercer TR, Thompson ER, Lakhani SR *et al*: **SNORD-host RNA Zfas1 is a regulator of mammary development and a potential marker for breast cancer**. *RNA* 2011, **17**(5):878-891.

2. Guil S, Esteller M: **Cis-acting noncoding RNAs: friends and foes**. *Nat Struct Mol Biol* 2012, **19**(11):1068-1075.

3. Villegas VE, Zaphiropoulos PG: **Neighboring gene regulation by antisense long non-coding RNAs**. *Int J Mol Sci* 2015, **16**(2):3251-3266.

4. Meyuhas O: **Chapter 1 Physiological Roles of Ribosomal Protein S6: One of Its Kind**. In: *International Review of Cell and Molecular Biology.* vol. Volume 268: Academic Press; 2008: 1-37.

Second revision

### Reviewer’s report 1: Chritine Vande Velde, Department of Neurosciences, University of Montreal

## Reviewer summary and comments

The authors have mostly addressed my previous concerns and is improved. However, that the ZFAS1 shRNA effect on rpS6 levels/phosphorylation is evident with only one of the two shRNA sequences attempted is a definite weakness of the manuscript. While the authors have explained there are technical limitations here, this data point is rather central to their final conclusion that ZFAS1 may represent a new class of lncRNAs which regulate their function. This caveat should be made obvious one page 13 and the discussion.

Authors’ response: *We have addressed this issue and now have replicated the effect of shRNA by using two independent shRNAs (BC1 and BC2). We have shown the data in Fig.* *6* *.*

**Minor comment**

The supplemental figures are presented out of order.

Authors’ response: *We have corrected the order for the Figures.*

### Reviewer’s report 2: Nicola Aceto, Department of Biomedicine, University of Basel

## Reviewer comments

(1) It is unclear to me what is the conclusion of the paragraph “Expression of ZFAS1 and ZNFX1 in breast cancer”. What do the authors conclude, other that ZFAS1 and ZNFX1 are expressed in both normal and cancer?

Authors’ response: *It is believed that many lncRNAs regulate closely linked protein-coding genes in cis. If this was the case with ZFAS1 and ZNFX1, it would be expected that the transcript abundance of the two genes should be correlated, either negatively (if ZNFX1 was suppressed by ZFAS1) or positively (if it was induced). The lack of any correlation is evidence that cis regulation involving this pair of genes does not apply. Such a conclusion provides a basis for seeking ZFAS1 effects at other locations and on other processes. Our report describes our search for alternative mechanisms, using an unbiased approach that did not rely on any preformed hypothesis.*

We have added “Many lncRNAs regulate the protein-coding genes in *cis*. If this was the case with *ZFAS1* and *ZNFX1*, it would be expected that the abundance of their transcripts should be related. The lack of any correlation is evidence that *cis* regulation involving this pair of genes does not apply, and provides a basis for seeking alternative *ZFAS1* activities” (page 8, lines 178-182).

Also in discussion added “Many antisense lncRNAs regulate the associated protein coding genes in *cis*. The lack of correlaton between *ZFAS1* and *ZNFX1* expression indicates that there is no apparent *cis* regulation between them*,* and provides a basis for seeking alternative *ZFAS1* activities” (page 15-16, lines 372-376).

## Reviewer comments

Additionally, expression differences between normal and basal or HER2-positive breast cancer, despite significant, are almost invisible. Same for ER- vs ER1. A statement at the end of this paragraph is needed to summarize what did we learn from these analyses.

Authors’ response: *Our earlier publication (2011), using a limited number of samples, presented preliminary evidence that ZFAS1 expression was down-regulated in breast cancer cells relative to normal breast epithelial cells. Our current study sought to investigate this potentially important finding more thoroughly, using the wealth of TCGA data now available. We have reported the results of our analysis, which found minimal differences between unselected neoplastic and normal breast samples, but which suggested that there may yet be subtle differences between ZFAS1 expression in certain subtypes of breast cancer and normal cells (Additional file* *2* *: Figure S2A). Thus our current analysis indicates that the original finding needs to be reinterpreted with caution. It leaves open the possibility that there may be subtype-specific effects on ZFAS1 regulation or activity.*

We have added “Our earlier publication (2011), using a limited number of samples, suggested that *ZFAS1* expression was down-regulated in breast cancer cells relative to normal breast epithelial cells. Our current study sought to investigate this finding more thoroughly, using large TCGA datasets, and found no differences between unselected neoplastic and normal breast samples. The possible subtle differences between *ZFAS1* expression in certain *subtypes* of breast cancer and normal cells (Additional file 2: Figure S2A) could reflect the large number of samples examined, and thus be of minimal clinical impact.*” (Page 9, lines 191-197).*

## Reviewer comments

(2) ZFAS1 and ZNFX1 primer efficiency: the authors have now included a second primer set to demonstrate that primer efficiency for ZFAS1 and ZNFX1 is similar, and the data is convincing. However, in the revised version of the manuscript they literally paste an excel sheet with lots of numbers as Additional file 13: Figure S10A and B. Clearly, “undigested” excel sheets are not meant to be pasted as figure panels, and it would be better to have a clearer figure that summarizes the data in a more compact form.

Authors’ response: *We provided the unprocessed data to satisfy the referee’s concerns regarding primer efficiency, but did not provide a more refined depiction of the data because such preliminary characterisations are routine and not usually published. However, we have replace Additional file* *13* *: Figure S10.*

## Reviewer comments

(3) I am still unconvinced about the explanation of the rationale for dissecting the biology of ZFAS1 in the context of normal vs neoplastic breast cells. The authors claim that ZFAS1 might be seen as a developmental gene (given its downregulation from pregnancy to lactation), and since many developmental genes are involved in cancer, it is worthwhile to investigate ZFAS1 in the context of cancer cells. This view is a bit simplistic and not very convincing at this stage, can the authors explain better and provide a stronger rationale that will help the reader understand the main reasons for investigating this gene?

Authors’ response: *We identified ZFAS1 in neoplastic and normal mammary epithelium, in a study that largely involved murine tissues. Because it is a novel transcript, and (for a lncRNA) very highly expressed, we sought to follow up this work in human neoplastic and normal breast cells as we did preliminary experiments in our previous paper in 2011. Here we extended the previous study and investigated functions of ZFAS1 in breast cancer cell lines. Indeed there are many reports that particular lncRNAs contribute to neoplastic behaviour (reviewed for breast cancer, reference 8). The association between developmental and cancer genes are also well accepted in the literature [1-4]. During the course of our work, others have reported investigations on ZFAS1 in different cell types (including cancer cells,) and have described mechanisms of action that do not seem to pertain to the cell type we are investigating. Our approach is thus an unbiased, methodical investigation of ZFAS1 activity in breast cells. We report that the data describing the relative expression in cancer and non-cancer tissues are more nuanced than first described. But there do appear to be differences between cancer and normal cells in ZFAS1 expression relative to that of coding genes associated with ribosomes (see the gradients of the regression lines Additional file* *3* *: Figure S3C, E, G and K). We anticipate that the significance of these differences will become apparent as ZFAS1 action is elucidated.*

## Reviewer comments

(4) P-S6 data in Fig. 6e/Additional file 8: Figure S7: revised data does not provide evidence that P-S6 nor total S6 are decreased as a consequence to ZFAS1 knockdown. First, it looks like bands in Fig. 6e have been cut/pasted from different experiments, therefore not suitable for publication. Second, the authors do not have a second independent ZFAS1 shRNA that shows a similar result, therefore off-target effects cannot be ruled out (BC3 clearly shows no difference in band density, strongly arguing that results obtained with BC2 are rather due to off-target effects). The authors are urged to either remove any claim on P-S6 and total S6, or to identify additional shRNAs that effectively suppresses ZFAS1 levels to test their hypotheses on P-S6.

Authors’ response: *We have provided a whole uncut immunoblot image to address the concern that Fig.* *6* *e was a composite (Additional file* *8* *: Figure S7 in previous submission). In the revised manuscript, we have included the full image of western blots for three shRNAs (BC1, BC2 and BC3). The differences between scrambled and knockdown shRNAs are also presented in the new version of Fig.* *6* *.*

We have added comments about shRNAs in page 14, line 325-330.

## Reviewer comments

(5) Accordingly to point 5, authors should remove all claims in the manuscript that are sustained by only one shRNA. In these cases, ZFAS1-specific effects cannot be distinguished from off-target effects.

Authors’ response: *We have performed the western blot analysis for a second shRNA BC1, and have shown significant down-regulation for phosphorylated RPS6. Also performed serum starvation and refeeding experiment for shRNA BC1 and confirmed similar result to shRNA BC2, Fig.* *6* *has been modified accordingly.*

### Reviewer’s report 3: Haruhiko Siomi, Department of Molecular Biology, Keio University

I will not describe the main achievements of the paper, since this was done in the review of the original submission. In the previous review, I was not convinced that ZFAS1 is associated with ribosomes.

On the whole it has been improved and the authors have experimentally addressed many of the reviewers concerns. However, I am still not convinced that ZFAS1 is associated with ribosomes. Additional file 1: Figure S1 shows that levels of ribosome binding to ZFAS1 transcripts are similar to those observed in background controls.

Authors’ response: *Additional file* *1* *: Figure S1 is based on ribosome profiling studies performed by Ingolia et al, a technique that involves digesting RNA and sequencing the portion bound to ribosomes to give a profile of ribosome occupancy. Therefore, it only represents RNAs that are being actively translated. As ZFAS1 is a non-coding RNA and is not translated, the lack of peaks in the ribosome profile is expected. It shows a similar profile to GAS5, a well-studied ribosome-associated long non-coding RNA. This has been addressed in Results section, “Protein Coding Potential of ZFAS1” and is quoted below:*

“The majority of the peaks corresponding to ribosomal occupancy overlapped with genomic regions of intron-derived snoRNAs. These peaks are a source of background RNA in profiling experiments, similar to that of *GAS5*, another lncRNA that is host to several snoRNAs as described by Ingolia et al” (page 7, lines 154-157).

## Reviewer’s comment

Figure 3a & b show that EDTA treatment, which dissociates ribosomes to ribosomal subunits, does not result in cosedimentation of ZFAS1 with large or small ribosomal subunits. Indeed, sedimentation profiles of ZFAS1 on sucrose density gradients with/without EDTA appear very similar, though the intensity of each band of ZFAS is reduced in B (with EDTA). Fig. 3d shows re-distribution of ZFAS1 from the “light polysomes” to the dissociated ribosomes. However, this re-distribution deserves band quantification and at least *n* = 3 to determine whether or not statistically sound. Comparing the distributions of GAPDH in Fig. 3b and d may lead one to wonder what the basis is for some of the major claims.

Figure 3a and b represent sucrose gradients without and with EDTA treatment, respectively. In this experiment, we asked whether *ZFAS1* associates with ribosomes in a manner that is similar to mRNAs. Upon EDTA treatment, the RNA associated with ribosomes would be released due to subunit dissociation. In Fig. 3c, we see the association of *ZFAS1* starting from fraction 15, peaking in fraction 23, and ending at fraction 28. These fractions would relate to 80S ribosomes and the left hand of the polysome peak in Fig. 3a. On the other hand, *GAPDH* association starts from fraction 23 and ends at 35, which relates to the polysome fractions in panel A. *ZFAS1* is found in fractions 10-23 in the EDTA-treated sample which relates to the ribosome subunits peak in panel B. *GAPDH* is found in similar fractions at higher abundance. A similar result was obtained in a second experiment and is presented in Additional file 15: Figure S12. These data confirmed the association of *ZFAS1* with ribosomes in a similar manner to *GAPDH* (mRNA), though *ZFAS1* appeared in lighter fractions. Since we cannot clearly define the 60S and 80S peaks in Fig. 3b, we used different approaches to separate the subunits and examine the association of *ZFAS1* with each subunit (Fig. 4).

We have treated samples with EDTA twice (data presented in Fig. 3 and Additional file 15: Figure S12). Comparing these results statistically is not possible, due to the nature of gradient collection, which inevitably produces variations in fraction numbers between different sucrose gradients. Therefore we have presented the results of both experiments in their entirely.

We also treated MDA-MB-468 cells with puromycin which serves as an acceptor of the growing peptide chain from the P-site, forming polypeptide-puromycin derivatives, and subsequently leading to premature termination and subunit dissociation. We treated cells with puromycin for different purposes, and our 30 min treatment did not achieve full subunit dissociation. Nevertheless, these conditions resulted in a partial redistribution of *ZFAS1* from heavier fractions to lighter ones (Additional file 16: Figure S13). The shift of *GAPDH* was more pronounced than that for *ZFAS1*, suggesting that *ZFAS1* release may need longer treatment. This experiment addresses our initial question, and shows that *ZFAS1* is associated with ribosomes, as is the mRNA for *GAPDH*, although it is found in 80S monosomes and in ligher polysomes.

## Reviewer’s comment

In Response to Reviewers, the authors state that “The graphs in Additional file 14: Figure S11 show ZFAS1 expression in fractions derived from the gradient.” However, Additional file 14: Figure S11 does not show such data. Additional file 15: Figure S12 shows that most ZFAS1 cosediments with fraction 17 but not with fraction 23/40S small ribosome subunits. If I understand right, 28S rRNA is not even associated with 60S fraction in their experiments!"

In our previous response we intended to refer to Additional file 15: Figure S12 not Figure S11. This was a typing mistake. In Additional file 14: Figure S11 (current version), red bars indicate 40S and 60S peaks. The arrow indicating the 60S peak for 28S rRNA had been positioned wrongly in the previous version (Additional file 15: Figure S12), and this has now been amended, confirming that 28S is indeed associated with the 60S fraction (Additional file 14: Figure S11). We thank the Reviewer for carefully perusing our manuscript and for alerting us to these errors.

Authors’ minor changes:

Additional file 5: Figure S4 is edited.

Figure S4-S7 in the previous revision is now presented as Figure Additional file 6: Figure S5; Additional file 7: Figure S6; Additional file 8: Figure S7 and Additional file 10: Figure S8.

Figure S8 in the previous revision is now deleted and the data presented in Fig. 6.

Figure S11 is deleted and data presented in Additional file 5: Figure S4-edited.

Table S1 and S2 are swapped in new version.

We have shortened the first paragraph of the discussion.

References:

1. Gailani, M.R. and A.E. Bale, *Developmental genes and cancer: role of patched in basal cell carcinoma of the skin.* J Natl Cancer Inst, 1997. **89**(15): p. 1103-9.

2. Ibsen, K.H. and W.H. Fishman, *Developmental gene expression in cancer.* Biochim Biophys Acta, 1979. **560**(2): p. 243-80.

3. Monk, M. and C. Holding, *Human embryonic genes re-expressed in cancer cells.* Oncogene, 2001. **20**(56): p. 8085-91.

4. Robson, E.J., S.J. He, and M.R. Eccles, *A PANorama of PAX genes in cancer and development.* Nat Rev Cancer, 2006. **6**(1): p. 52-62.

5. Askarian-Amiri, M.E., et al., *SNORD-host RNA Zfas1 is a regulator of mammary development and a potential marker for breast cancer.* RNA, 2011. **17**(5): p. 878-91.

## Additional files

Additional file 1: Figure S1.
*ZFAS1* is unlikely to encode a protein. Ribosome occupancy derived from multiple ribosome profiling studies according to the GWIPS database is mapped to *ZFAS1*. Red peaks from ribosome profile indicate the level of ribosome occupancy whereas green peaks from mRNA seq coverage indicate the level of transcription of a particular gene region. *ZFAS1* is indicated in blue, with numbers indicating nucleotide number for each exon above the gene layout. Potential open reading frames, shown in pink, were predicted using ExPASy and mapped to the genomic layout of *ZFAS1.* Peaks corresponding to ribosome occupancy were then overlaid with ORFs, with the peaks mapping to snoRNAs in the intronic regions of *ZFAS1. (PDF 271 kb)*

Additional file 2: Figure S2. Analyses of *ZFAS1* in breast cancer samples derived from TCGA. A(i) Expression of *ZFAS1* in normal breast (*n* = 113) and breast cancer (*n* = 1069) samples. (ii) Expression of *ZFAS1* by tumour subtype based on PAM50 classification. *ZFAS1* is more highly expressed in normal tissues compared to basal and HER2 breast cancer subtypes. (iii) Expression of *ZFAS1* in ER+ (*n* = 601) and ER- (*n* = 179) breast cancer samples. Unpaired Student’s t-test showed that *ZFAS1* was differentially expressed according to estrogen status. (B) Kaplan-Meier plot generated from http://www.oncolnc.org/ of TCGA breast cancer data set. High expressers are those 50 % of patients with the highest *ZFAS1* expression, and low expressers are those 50 % of patients with the lowest *ZFAS1* expression. High expressers of *ZFAS1* do not show altered survival up to 6000 days. (C) Gene expression of candidate ribosomal proteins by tumour subtype based on PAM50 classification. Unpaired student’s t-test relative to normal tissue samples was used to calculate P values. (PDF 475 kb)
Additional file 3: Figure S3. Expression of *ZFAS1* in breast cancer tissue derived from TCGA. (A) Expression of *ZFAS1* in tumour and non-tumour samples. (B-K) Expression of concordantly regulated ribosomal protein genes in breast cancer and normal breast tissue (TCGA data). Correlation of the abundances of these gene transcripts to that of *ZFAS1* is plotted in the right panels for normal breast tissue and breast cancer samples. Student’s t test was used to determine the significance of difference in expression between tumour (T) and non-tumour (N) samples, *, **, *** represent p values of >0.05, >0.005 and >0.001 respectively. (PDF 596 kb)
Additional file 4: Table S1: Correlation of *ZFAS1* expression with that of randomly selected genes in human (i) non-tumour and (ii) breast cancer samples (TCGA data). (DOC 38 kb)
Additional file 5: Figure S4. Effect of *ZFAS1* knockdown on cell phenotype. (A) Proliferation rates of cells transfected with vectors expressing control (scrambled RNA) and *ZFAS1*-specific shRNA BC2 and BC3 as determined by SRB assay. Error bars are SEM, *n* =2. (B) Size of cells expressing control (scrambled) and *ZFAS1*-specific shRNA BC2 was determined using a Coulter electronic particle counter. (C) Nascent protein synthesis as quantified by uptake of the fluorescent amino acid analogue, Click-iT® AHA, in *ZFAS1* knockdown BC2 and scrambled control cells, *n* = 2. (PDF 451 kb)
Additional file 6: Figure S5.
*ZFAS1* has a 5’TOP sequence and may resist NMD. (A) Sequence of the 5’ region of human *ZFAS1* as determined by 5’RACE aligned against *ZFAS1,* variant 4 from NCBI136/hg138 assembly. (B) Effect of serum starvation on the abundance of *ZFAS1* and *GAS5*. Different cell lines were used to examine the effect of serum starvation for up to 72 h. qPCR was performed using total RNA extracted from each cell to measure the level of *ZFAS1* and *GAS5. 18S * and 28S rRNA transcripts were used to normalise the expression of *ZFAS1* and *GAS5*. Fold change relative to time 0 is shown on the Y axis, and treatment time (h) shown on the X axis. Error bars are SEM of three biological replicates, p values were calculated using Student’s t test. (PDF 471 kb)
Additional file 7: Figure S6. Effect of puromycin, a translational inhibitor, on the abundance of *ZFAS1* and *GAS5*. Relative expression of genes was measured by qPCR using total RNA extracted from each cell. *18S* and *28S* rRNA transcripts were used to normalise the expression of *ZFAS1* and *GAS5*. The Y axis represents the fold change relative to time 0. The X axis shows treatment time. Error bars are SEM of three biological replicates, p values were calculated using Student’s t test. (PDF 172 kb)
Additional file 8: Figure S7. Effect of cycloheximide, a translational inhibitor, on the abundance of *ZFAS1* and *GAS5*. Relative expression of genes was measured by qPCR using total RNA extracted from each cell. *18S* and *28S* rRNA transcripts were used to normalise the expression of *ZFAS1* and *GAS5*. The Y axis represents the fold change relative to time 0. The X axis shows treatment time. Error bars are SEM of three biological replicates, p values were calculated using Student’s t test. (PDF 136 kb)
Additional file 9: Table S2. List of primers used in these experiments. The sequences are 5′ to 3′. (DOC 43 kb)
Additional file 10: Figure S8.
*ZFAS1* is associated with actively translating ribosomes in an isoform-independent manner. (A) Polysome distribution of MDA-MB-468 cell lysates as shown in Fig. 3. Fractions from the top of the gradient to the bottom are shown from left to right on the X axis. Fractions were collected in 36 equal volumes, of which every third was used for RNA extraction, and cDNA synthesised for PCR. The presence of *ZFAS1* expression was assessed using primers E1F3-E5R1, with *GAPDH* acting as a positive control. The presence of three bands confirms at least 4 out of five isoforms are present in each fraction. (PDF 1333 kb)
Additional file 11: Figure S9. Genomic orientation of *ZFAS1* and four shRNA used in these experiments. (PDF 127 kb)
Additional file 12: Table S3. List of sequences of shRNA used to target exon 5 of *ZFAS1*. (DOC 27 kb)
Additional file 13: Figure S10. Primer efficiency of *ZFAS1* and *ZNFX1.* A) The primer efficiency test for the primers used in the experiments. B) Slopes of standard curve indicate PCR efficiency for *ZFAS1* and *ZNFX1* primers sets. The X axis represent the log of dilution. Y axis shows Ct values. C) Correlation between the expression of *ZFAS1* and *ZNFX1* in two different primer sets in panel of cell lines (PDF 317 kb)
Additional file 14: Figure S11. The presence of *ZFAS1, 18S* and *28S* expression were assessed by qPCR using fractions derived from dissociated ribosomes (Fig. 4i). Red bars show the samples derived from the peak of the graph in Fig. 4Ai for 40S and 60S subunit. (PDF 235 kb)
Additional file 15: Figure S12.
*ZFAS1* is associated with actively translating ribosomes. (A) Polysome distribution of MDA-MB-468 cell lysates as separated on a 7–47 % sucrose gradient. Absorbance at 260 nm is shown on the Y axis. Fractions from the top of the gradient to the bottom are shown from left to right on the X axis. Fractions were collected in 36 equal volumes, of which every third was used for RNA extraction, and cDNA synthesised for PCR. (B) Polysome distribution of MDA-MB-468 cell lysate separated on a 7–47 % sucrose gradient containing EDTA instead of MgCl_2_. Loss of the polysome peak is observed, together with a leftward shift of the ribosome subunits. (C) and (D) Quantitative expression of *ZFAS1* and *GAPDH* measured by qPCR relative to *18S* and *28S* rRNAs prepared with and without the addition of EDTA. Arrows indicate where ribosomal features are observed on profiles in relation to fraction number. (PDF 536 kb)
Additional file 16: Figure S13.
*ZFAS1* is associated with actively translating ribosomes. (A and B) Polysome distribution of MDA-MB-468 cell lysate derived from cells treated with and without puromycin as separated on a 7–47 % sucrose gradient. Absorbance at 260 nm is shown on the Y axis. Fractions from the top of the gradient to the bottom are shown from left to right on the X axis. Fractions were collected in 36 equal volumes, of which every third was used for RNA extraction, and cDNA synthesised for PCR. (C and D) Quantitative expression of *ZFAS1* measured by qPCR from fractions collected from 7–47 % sucrose gradient. The result present the abundance of *ZFAS1* in control (untreated) and cells treated with puromycin for 30 min. *ZFAS1* peak is shifted toward left in puromycin treated cells. (E and F) Quantitative expression of *GAPDH* measured by qPCR, from fractions collected from 7–47 % sucrose gradient. The result present the abundance of *GAPDH* in control (untreated) and cells treated with puromycin for 30 min. *GAPDH* peak is shifted toward left in puromycin treated cells. Left and right boxes are presenting results from two independent experiments. (PDF 248 kb)

## Abbreviations

FCS: Foetal Calf Serum
GAPDH: GlycerAldehyde 3-Phosphate DeHydrogenase
LncRNA: Long Non Coding RNA
mlonRNAs: MRNA-type LOng non-coding RNAs
NEAT1: Nuclear Enriched Abundant Transcript 1
NMD: Nonsense Mediated Decay
ORF: Open Reading Frame
snoRNA: Small NucleOlar RNA
*Uchl1AS*: Ubiquitin Carboxy-terminal Hydrolase L1, AntiSense
ZFAS1: ZNFX1 Antisense RNA 1
ZNFX1: Zinc Finger, NFX1-type containing 1
