# Supplementary figures and images for: ZFAS1: a long noncoding RNA associated with ribosomes in breast cancer cells
Source: Biol Direct. 2016 Nov 21;11:62. doi: 10.1186/s13062-016-0165-y (PMC5117590; doi:10.1186/s13062-016-0165-y)

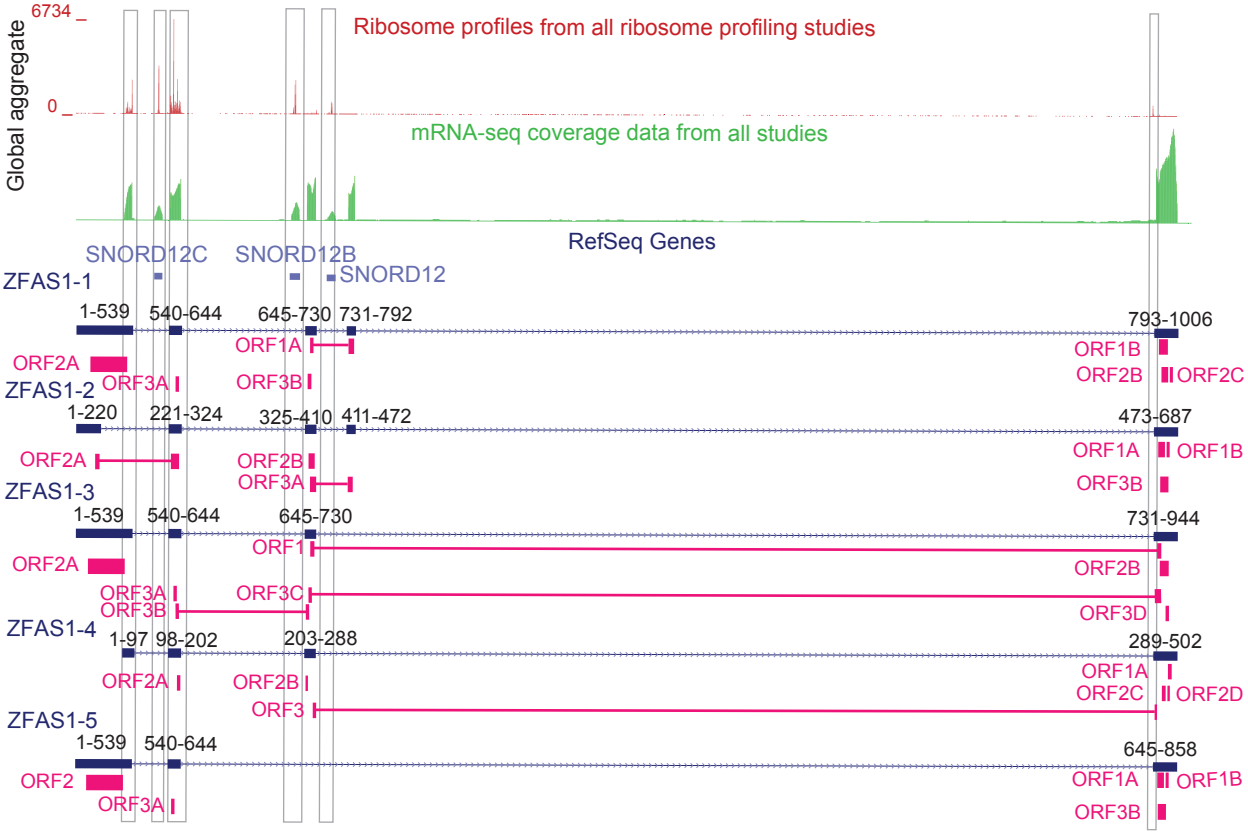

Supplement: Additional file 1: Figure S1. — ZFAS1 is unlikely to encode a protein. Ribosome occupancy derived from multiple ribosome profiling studies according to the GWIPS database is mapped to ZFAS1. Red peaks from ribosome profile indicate the level of ribosome occupancy whereas green peaks from mRNA seq coverage indicate the level of transcription of a particular gene region. ZFAS1 is indicated in blue, with numbers indicating nucleotide number for each exon above the gene layout. Potential open reading frames, shown in pink, were predicted using ExPASy and mapped to the genomic layout of ZFAS1. Peaks corresponding to ribosome occupancy were then overlaid with ORFs, with the peaks mapping to snoRNAs in the intronic regions of ZFAS1. (PDF 271 kb) [file 13062_2016_165_MOESM1_ESM.pdf]

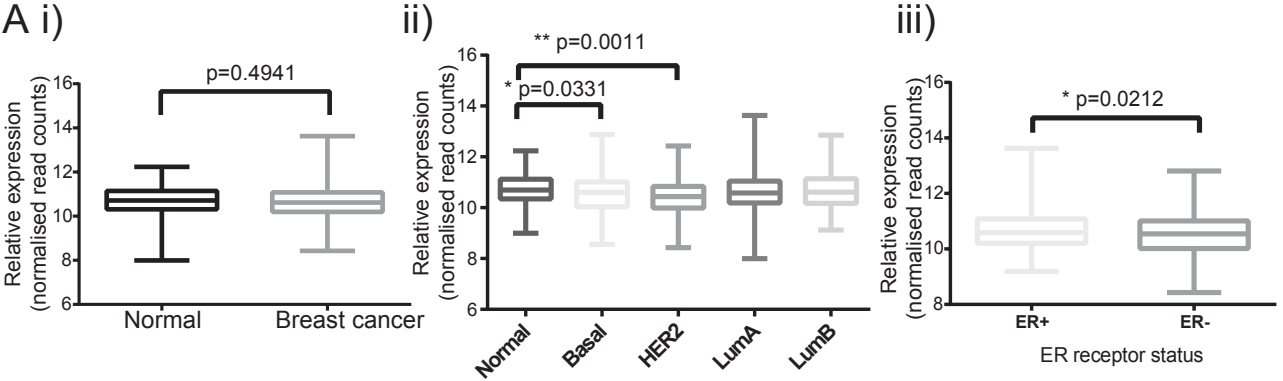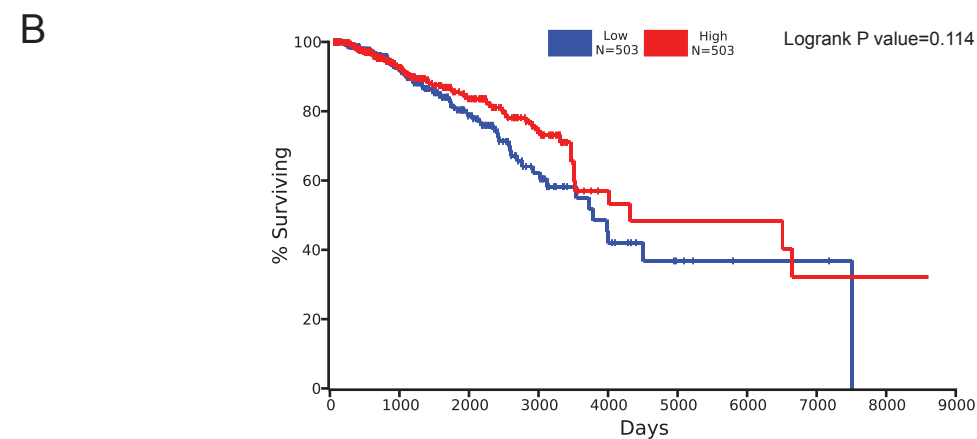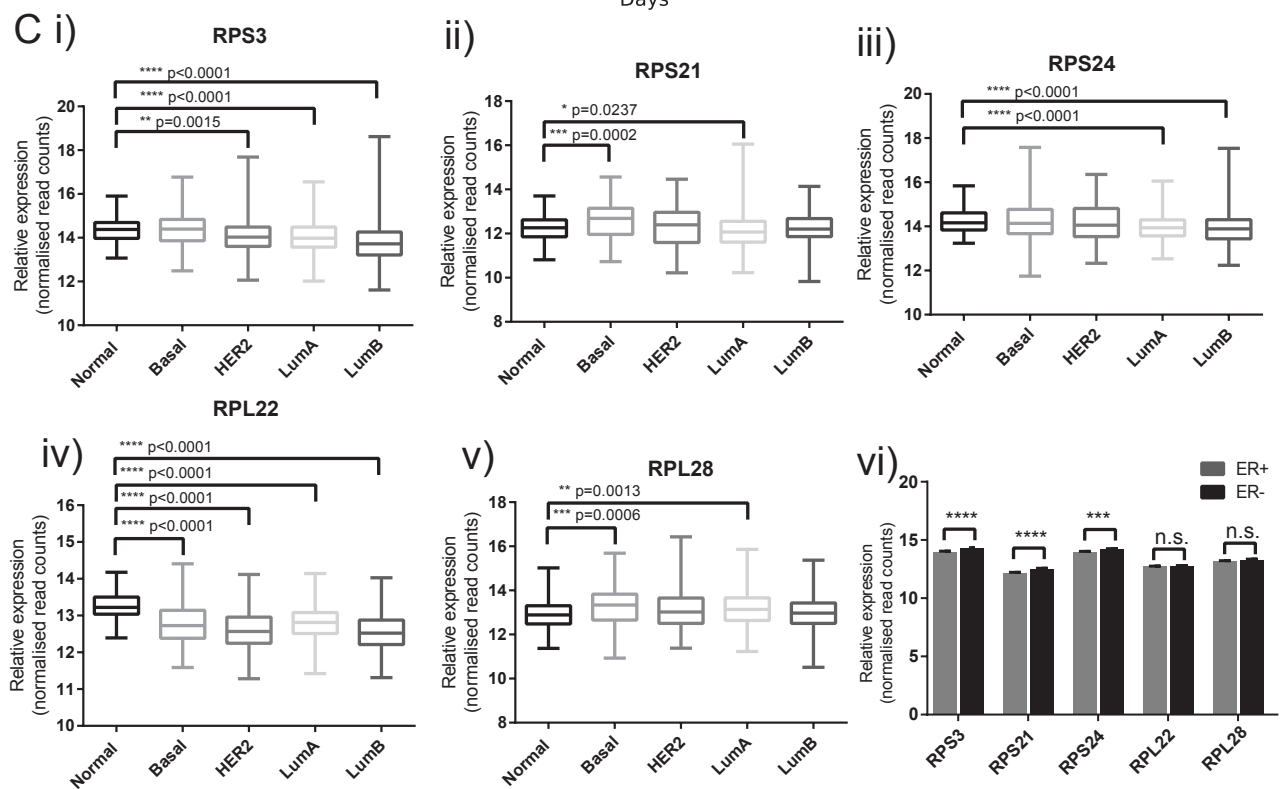

Supplement: Additional file 2: Figure S2. — Analyses of ZFAS1 in breast cancer samples derived from TCGA. A(i) Expression of ZFAS1 in normal breast (n = 113) and breast cancer (n = 1069) samples. (ii) Expression of ZFAS1 by tumour subtype based on PAM50 classification. ZFAS1 is more highly expressed in normal tissues compared to basal and HER2 breast cancer subtypes. (iii) Expression of ZFAS1 in ER+ (n = 601) and ER- (n = 179) breast cancer samples. Unpaired Student’s t-test showed that ZFAS1 was differentially expressed according to estrogen status. (B) Kaplan-Meier plot generated from http://www.oncolnc.org/ of TCGA breast cancer data set. High expressers are those 50 % of patients with the highest ZFAS1 expression, and low expressers are those 50 % of patients with the lowest ZFAS1 expression. High expressers of ZFAS1 do not show altered survival up to 6000 days. (C) Gene expression of candidate ribosomal proteins by tumour subtype based on PAM50 classification. Unpaired student’s t-test relative to normal tissue samples was used to calculate P values. (PDF 475 kb) [file 13062_2016_165_MOESM2_ESM.pdf]

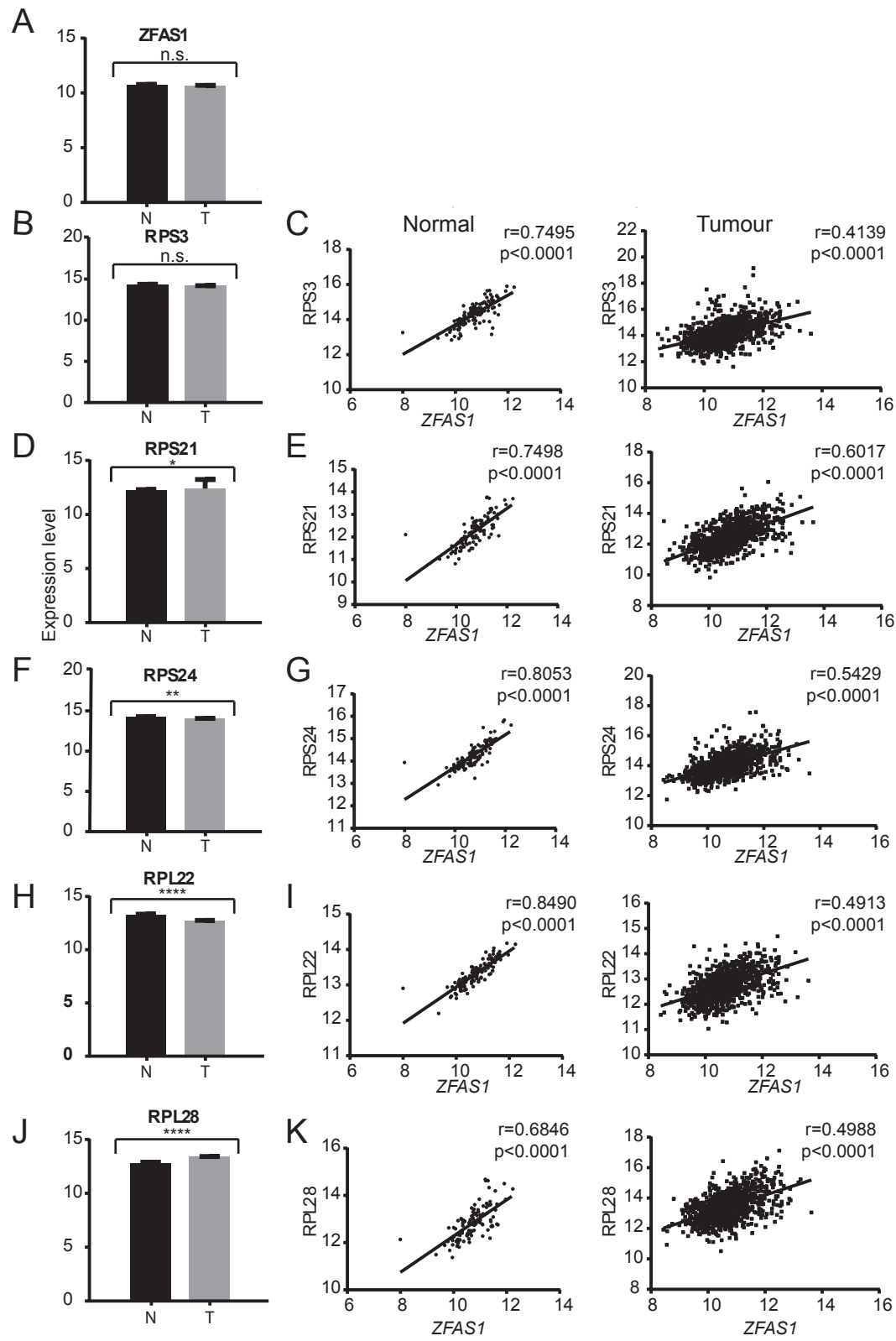

Supplement: Additional file 3: Figure S3. — Expression of ZFAS1 in breast cancer tissue derived from TCGA. (A) Expression of ZFAS1 in tumour and non-tumour samples. (B-K) Expression of concordantly regulated ribosomal protein genes in breast cancer and normal breast tissue (TCGA data). Correlation of the abundances of these gene transcripts to that of ZFAS1 is plotted in the right panels for normal breast tissue and breast cancer samples. Student’s t test was used to determine the significance of difference in expression between tumour (T) and non-tumour (N) samples, *, **, *** represent p values of >0.05, >0.005 and >0.001 respectively. (PDF 596 kb) [file 13062_2016_165_MOESM3_ESM.pdf]

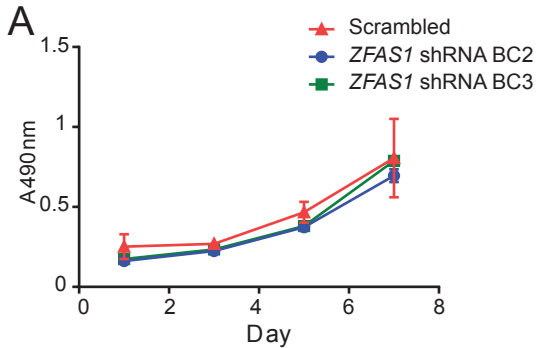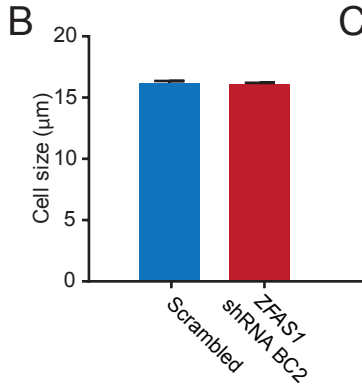

Supplement: Additional file 5: Figure S4. — Effect of ZFAS1 knockdown on cell phenotype. (A) Proliferation rates of cells transfected with vectors expressing control (scrambled RNA) and ZFAS1-specific shRNA BC2 and BC3 as determined by SRB assay. Error bars are SEM, n =2. (B) Size of cells expressing control (scrambled) and ZFAS1-specific shRNA BC2 was determined using a Coulter electronic particle counter. (C) Nascent protein synthesis as quantified by uptake of the fluorescent amino acid analogue, Click-iT® AHA, in ZFAS1 knockdown BC2 and scrambled control cells, n = 2. (PDF 451 kb) [file 13062_2016_165_MOESM5_ESM.pdf]

A

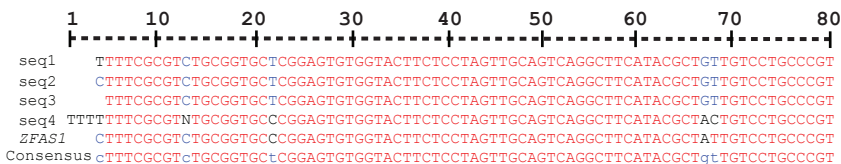

B

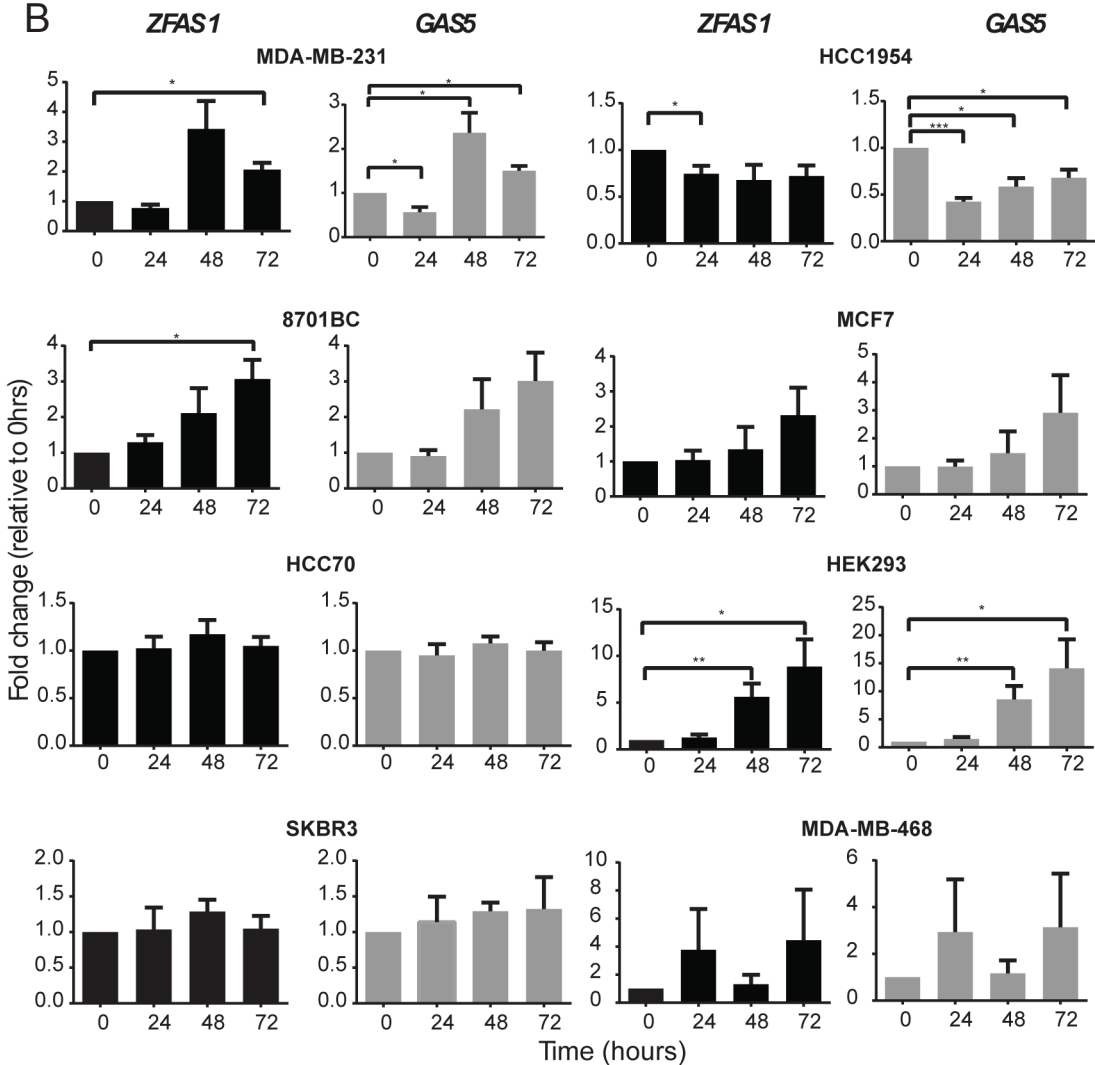

Supplement: Additional file 6: Figure S5. — ZFAS1 has a 5’TOP sequence and may resist NMD. (A) Sequence of the 5’ region of human ZFAS1 as determined by 5’RACE aligned against ZFAS1, variant 4 from NCBI136/hg138 assembly. (B) Effect of serum starvation on the abundance of ZFAS1 and GAS5. Different cell lines were used to examine the effect of serum starvation for up to 72 h. qPCR was performed using total RNA extracted from each cell to measure the level of ZFAS1 and GAS5. 18S and 28S rRNA transcripts were used to normalise the expression of ZFAS1 and GAS5. Fold change relative to time 0 is shown on the Y axis, and treatment time (h) shown on the X axis. Error bars are SEM of three biological replicates, p values were calculated using Student’s t test. (PDF 471 kb) [file 13062_2016_165_MOESM6_ESM.pdf]

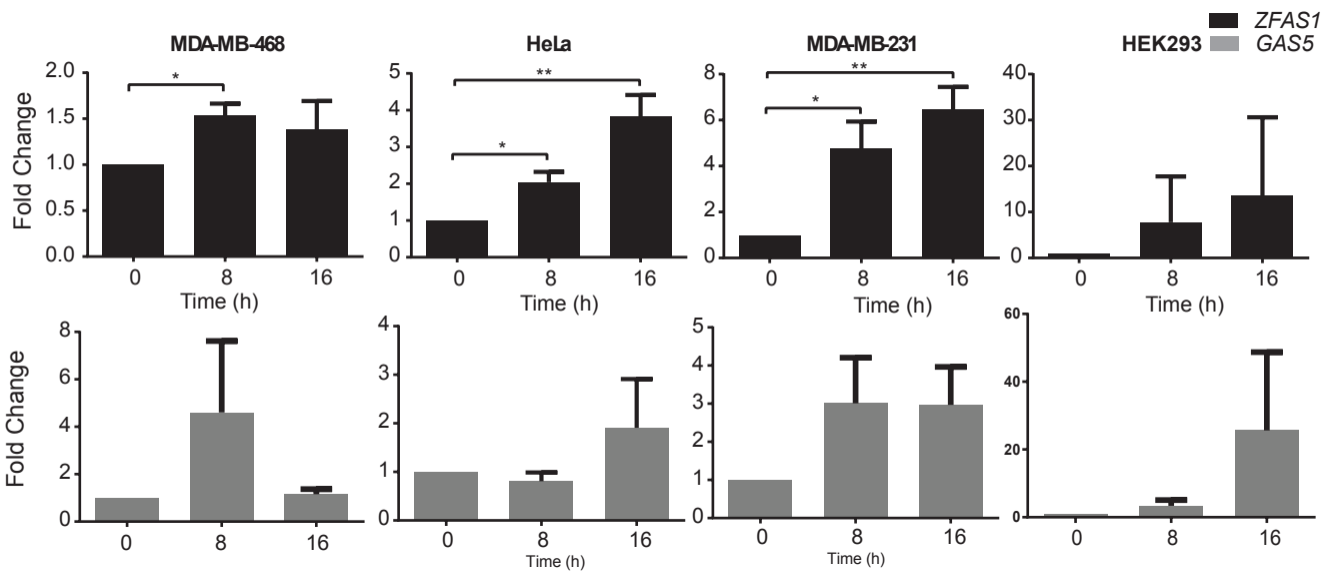

Supplement: Additional file 7: Figure S6. — Effect of puromycin, a translational inhibitor, on the abundance of ZFAS1 and GAS5. Relative expression of genes was measured by qPCR using total RNA extracted from each cell. 18S and 28S rRNA transcripts were used to normalise the expression of ZFAS1 and GAS5. The Y axis represents the fold change relative to time 0. The X axis shows treatment time. Error bars are SEM of three biological replicates, p values were calculated using Student’s t test. (PDF 172 kb) [file 13062_2016_165_MOESM7_ESM.pdf]

**ZFAS1****GAS5**

MDA-MB-468

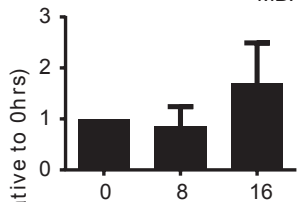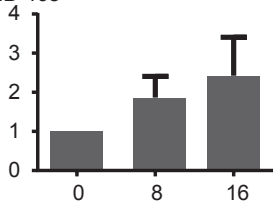

HeLa

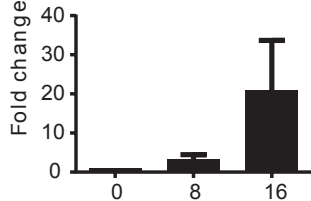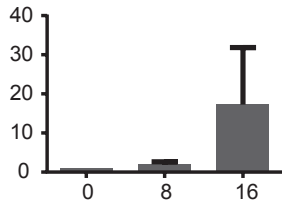

Time (hours)

Supplement: Additional file 8: Figure S7. — Effect of cycloheximide, a translational inhibitor, on the abundance of ZFAS1 and GAS5. Relative expression of genes was measured by qPCR using total RNA extracted from each cell. 18S and 28S rRNA transcripts were used to normalise the expression of ZFAS1 and GAS5. The Y axis represents the fold change relative to time 0. The X axis shows treatment time. Error bars are SEM of three biological replicates, p values were calculated using Student’s t test. (PDF 136 kb) [file 13062_2016_165_MOESM8_ESM.pdf]

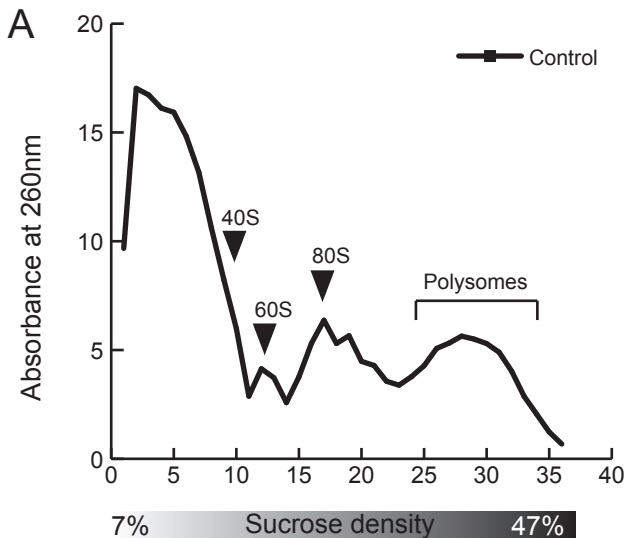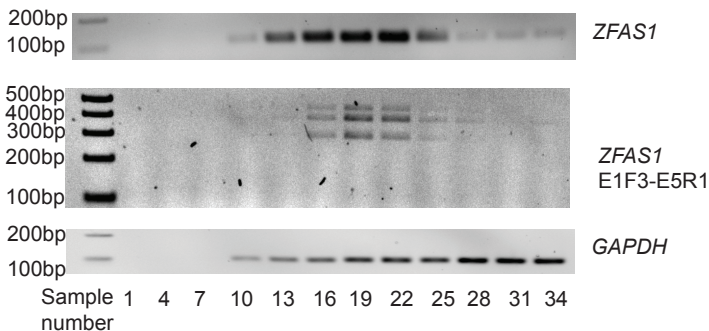

Supplement: Additional file 10: Figure S8. — ZFAS1 is associated with actively translating ribosomes in an isoform-independent manner. (A) Polysome distribution of MDA-MB-468 cell lysates as shown in Fig. 3. Fractions from the top of the gradient to the bottom are shown from left to right on the X axis. Fractions were collected in 36 equal volumes, of which every third was used for RNA extraction, and cDNA synthesised for PCR. The presence of ZFAS1 expression was assessed using primers E1F3-E5R1, with GAPDH acting as a positive control. The presence of three bands confirms at least 4 out of five isoforms are present in each fraction. (PDF 1333 kb) [file 13062_2016_165_MOESM10_ESM.pdf]

ZFAS1

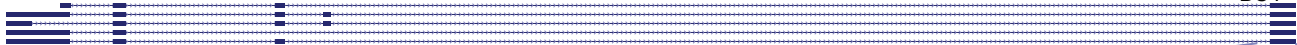

BC1  
BC2  
BC3  
BC4

BC1

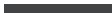

BC2

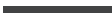

BC3

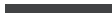

BC4

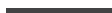

ZFAS1

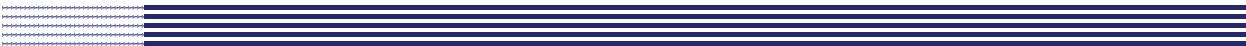

Supplement: Additional file 11: Figure S9. — Genomic orientation of ZFAS1 and four shRNA used in these experiments. (PDF 127 kb) [file 13062_2016_165_MOESM11_ESM.pdf]

# ZFAS1

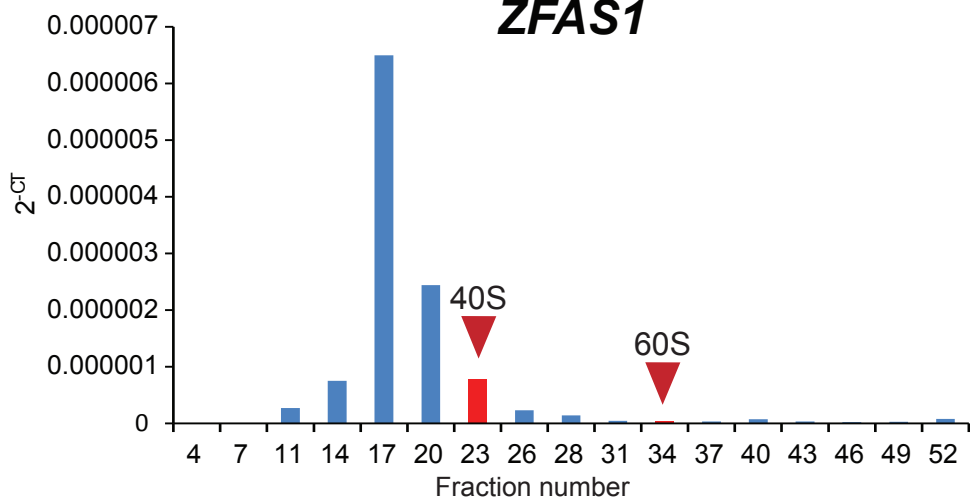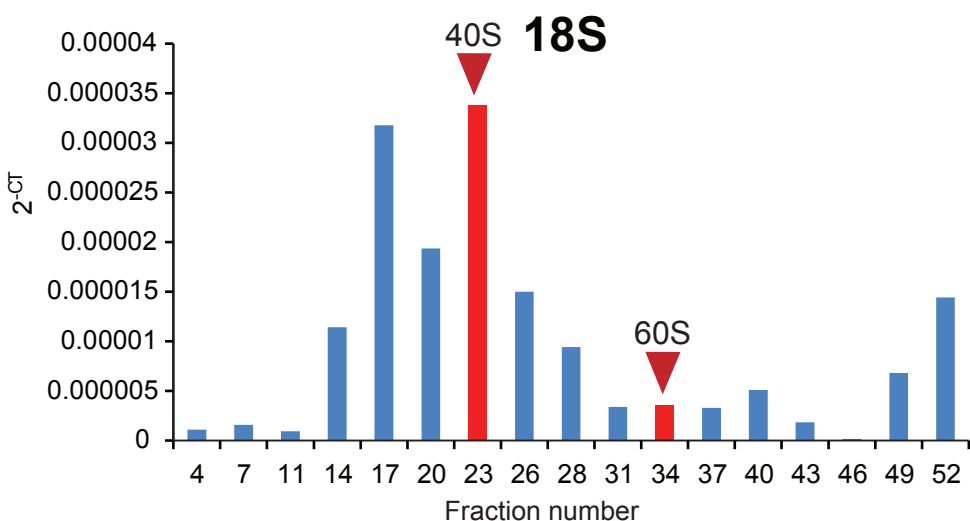

# 28S

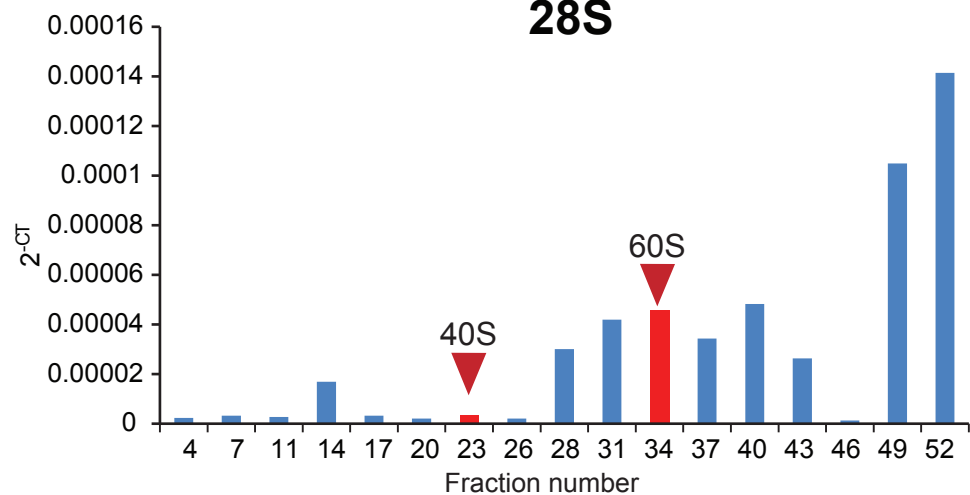

Supplement: Additional file 14: Figure S11. — The presence of ZFAS1, 18S and 28S expression were assessed by qPCR using fractions derived from dissociated ribosomes (Fig. 4i). Red bars show the samples derived from the peak of the graph in Fig. 4Ai for 40S and 60S subunit. (PDF 235 kb) [file 13062_2016_165_MOESM14_ESM.pdf]

**A**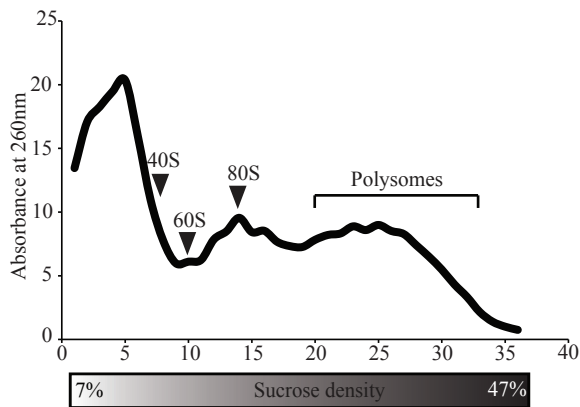**B**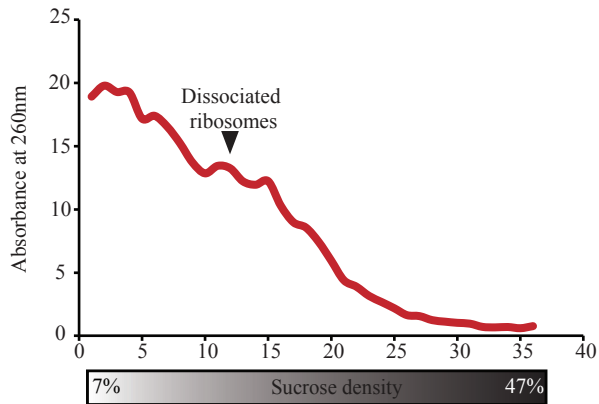**C**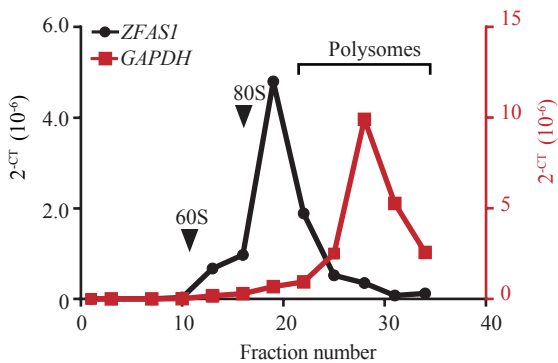**D**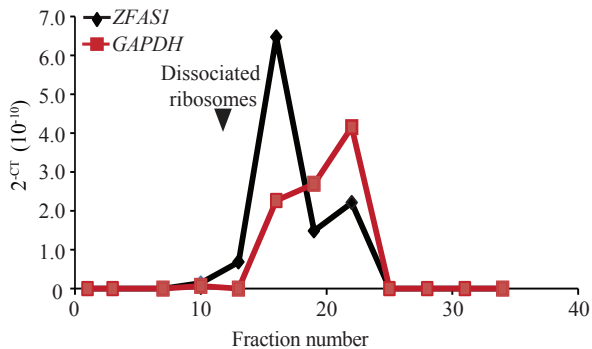

Supplement: Additional file 15: Figure S12. — ZFAS1 is associated with actively translating ribosomes. (A) Polysome distribution of MDA-MB-468 cell lysates as separated on a 7–47 % sucrose gradient. Absorbance at 260 nm is shown on the Y axis. Fractions from the top of the gradient to the bottom are shown from left to right on the X axis. Fractions were collected in 36 equal volumes, of which every third was used for RNA extraction, and cDNA synthesised for PCR. (B) Polysome distribution of MDA-MB-468 cell lysate separated on a 7–47 % sucrose gradient containing EDTA instead of MgCl2. Loss of the polysome peak is observed, together with a leftward shift of the ribosome subunits. (C) and (D) Quantitative expression of ZFAS1 and GAPDH measured by qPCR relative to 18S and 28S rRNAs prepared with and without the addition of EDTA. Arrows indicate where ribosomal features are observed on profiles in relation to fraction number. (PDF 536 kb) [file 13062_2016_165_MOESM15_ESM.pdf]

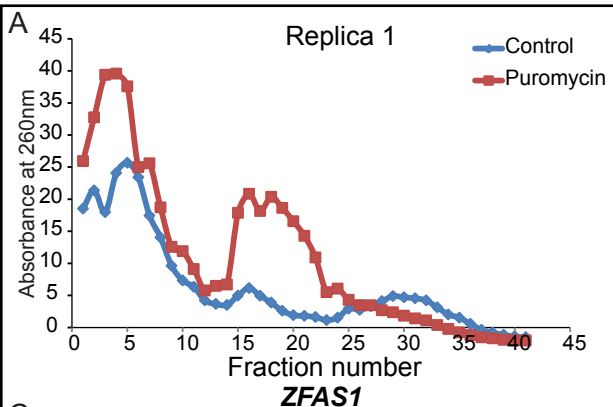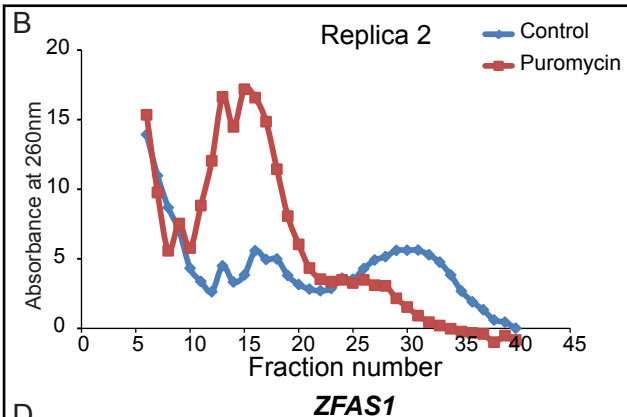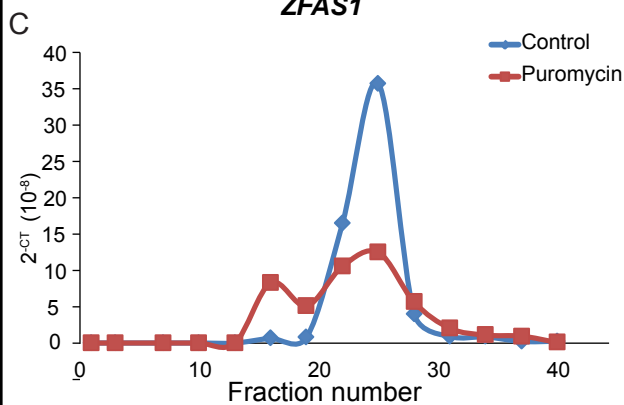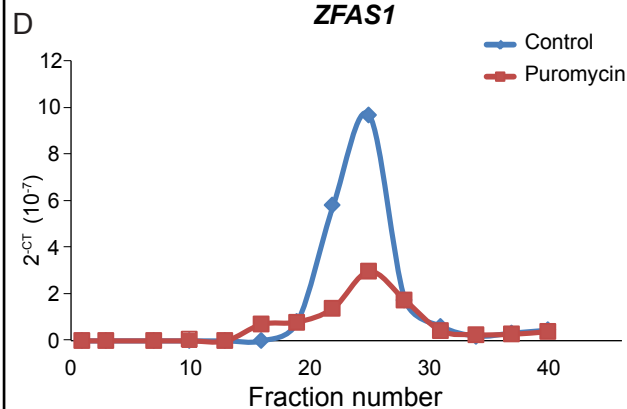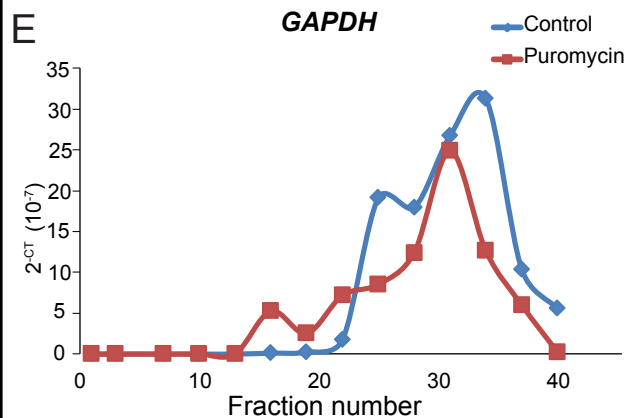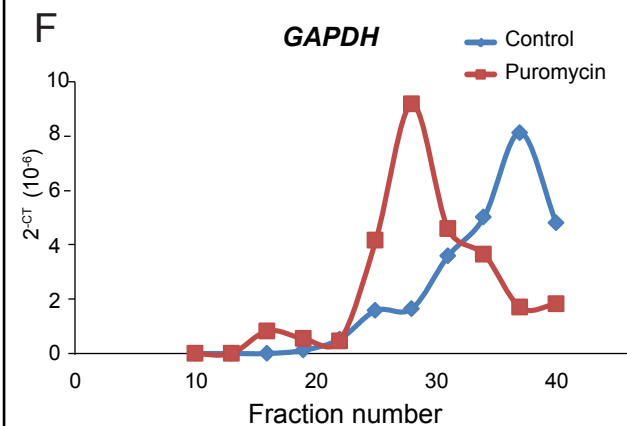

Supplement: Additional file 16: Figure S13. — ZFAS1 is associated with actively translating ribosomes. (A and B) Polysome distribution of MDA-MB-468 cell lysate derived from cells treated with and without puromycin as separated on a 7–47 % sucrose gradient. Absorbance at 260 nm is shown on the Y axis. Fractions from the top of the gradient to the bottom are shown from left to right on the X axis. Fractions were collected in 36 equal volumes, of which every third was used for RNA extraction, and cDNA synthesised for PCR. (C and D) Quantitative expression of ZFAS1 measured by qPCR from fractions collected from 7–47 % sucrose gradient. The result present the abundance of ZFAS1 in control (untreated) and cells treated with puromycin for 30 min. ZFAS1 peak is shifted toward left in puromycin treated cells. (E and F) Quantitative expression of GAPDH measured by qPCR, from fractions collected from 7–47 % sucrose gradient. The result present the abundance of GAPDH in control (untreated) and cells treated with puromycin for 30 min. GAPDH peak is shifted toward left in puromycin treated cells. Left and right boxes are presenting results from two independent experiments. (PDF 248 kb) [file 13062_2016_165_MOESM16_ESM.pdf]
