# Supplementary material for: ZFAS1: a long noncoding RNA associated with ribosomes in breast cancer cells
Source: Biol Direct. 2016 Nov 21;11:62. doi: 10.1186/s13062-016-0165-y (PMC5117590; doi:10.1186/s13062-016-0165-y)
Supplement: Additional file 4: Table S1: — Correlation of ZFAS1 expression with that of randomly selected genes in human (i) non-tumour and (ii) breast cancer samples (TCGA data). (DOC 38 kb) [file 13062_2016_165_MOESM4_ESM.doc]

**Supplementary Table 1: Correlation of *ZFAS1* expression with that of randomly selected genes in human (i) non-tumour and (ii) breast cancer samples (TCGA data).**

| **Solid tissue normal** | | | | | | |
| --- | --- | --- | --- | --- | --- | --- |
|  | **ZFAS1 vs. RAI14** | **ZFAS1 vs. MFSD1** | **ZFAS1 vs. PIK3R5** | **ZFAS1 vs. FERMT2** | **ZFAS1 vs. TUBGCP6** | **ZFAS1 vs. MRPL16** |
| **Pearson r** | 0.1049 | 0.1769 | -0.223 | -0.223 | 0.1851 | -0.1178 |
| **P (two-tailed)** | 0.271 | 0.062 | 0.0181 | 0.0181 | 0.0508 | 0.214 |
| **P value summary** | ns | ns | * | * | ns | ns |
| **Significant? (alpha = 0.05)** | No | No | Yes | Yes | No | No |
| **Number of XY Pairs** | 113 | 113 | 113 | 113 | 113 | 113 |

| **Tumour** | | | | | | |
| --- | --- | --- | --- | --- | --- | --- |
|  | **ZFAS1 vs. RAI14** | **ZFAS1 vs. MFSD1** | **ZFAS1 vs. PIK3R5** | **ZFAS1 vs. FERMT2** | **ZFAS1 vs. TUBGCP6** | **ZFAS1 vs. MRPL16** |
| **Pearson r** | -0.1297 | -0.2133 | -0.05938 | -0.05938 | -0.01216 | 0.2074 |
| **P (two-tailed)** | < 0.0001 | < 0.0001 | 0.0523 | 0.0523 | 0.6913 | < 0.0001 |
| **P value summary** | **** | **** | ns | ns | ns | **** |
| **Significant? (alpha = 0.05)** | Yes | Yes | No | No | No | Yes |
| **Number of XY Pairs** | 1049 | 1049 | 1049 | 1049 | 1049 | 1049 |
