# Supplementary material for: ZFAS1: a long noncoding RNA associated with ribosomes in breast cancer cells
Source: Biol Direct. 2016 Nov 21;11:62. doi: 10.1186/s13062-016-0165-y (PMC5117590; doi:10.1186/s13062-016-0165-y)
Supplement: Additional file 9: Table S2. — List of primers used in these experiments. The sequences are 5′ to 3′. (DOC 43 kb) [file 13062_2016_165_MOESM9_ESM.doc]

Supplementary Table 2: List of primers used in these experiments. The sequences are 5’ to 3’.

| **Primer name** | **Primer sequence** |
| --- | --- |
| **Isoform detection** |  |
| ZFAS1 E1F1 | CAGGGTGGAGAGCACGAG |
| ZFAS1 E1F2 | GAGATGACTGCGCCCAAG |
| ZFAS1 E1F3 | TTGCAGTCAGGCTTCATACG |
| ZFAS1 E5R1 | GAAGTGGGCACAGCCTTTTA |
| ZFAS1 E2R1 | GTACCCGCTGGCTGCTCTAA |
| ZFAS1 E2R2 | AGATGTCTGCACGTGGCTTC |
|  |  |
| **qPCR** |  |
| ZFAS1 E2-F | AAGCCACGTGCAGACATCTA |
| ZFAS1E3-R | CTACTTCCAACACCCGCATT |
| ZNFX1-F | ATCATGCTTTGGACCAGTTTCT |
| ZNFX1-R | TAGGGTGAACTGCTTCAGGATT |
| 18S-F | GTAACCCGTTGAACCCCATT |
| 18S-R | CCATCCAATCGGTAGTAGCG |
| 28S-F | CCATGGAAGTCGGAATCC |
| 28S-R | AGCGCCATCCATTTTCAG |
| HPRT-F | TGAGGATTTGGAAAGGGTGT |
| HPRT-R | GCACACAGAGGGCTACAATG |
| GAPDH-F | ACGGG AAGCTTGTCATCAAT |
| GAPDH-R | TGGACTCCACGACGTACTCA |
|  |  |
| **5’RACE** |  |
| AN poly C | GCATGCGCGCGGCCGCGG AGGCCCCCC CCCCCCCC |
| AN | GCATGCGCGCGGCCGCGG AGG |
| ZFAS1 E2R1 | GTACCCGCTGGCTGCTCTAA |
| ZFAS1 E2R2 | AGATGTCTGCACGTGGCTTC |
