# Supplementary material for: ZFAS1: a long noncoding RNA associated with ribosomes in breast cancer cells
Source: Biol Direct. 2016 Nov 21;11:62. doi: 10.1186/s13062-016-0165-y (PMC5117590; doi:10.1186/s13062-016-0165-y)
Supplement: Additional file 12: Table S3. — List of sequences of shRNA used to target exon 5 of ZFAS1. (DOC 27 kb) [file 13062_2016_165_MOESM12_ESM.doc]

Supplementary Table 3: List of sequences of shRNA used to target exon 5 of *ZFAS1*.

| **No.** | **Target sequence(DNA)** |
| --- | --- |
| BC1 | GCTTTCATGAAAGTGAAGATC |
| BC2 | GAACCAGTTCCACAAGGTTAC |
| BC3 | GGCTGTGCCCACTTCAAGAAT |
| BC4 | GCCCACTTCAAGAATGTCATT |
