# Supplementary material for: ZFAS1: a long noncoding RNA associated with ribosomes in breast cancer cells
Source: Biol Direct. 2016 Nov 21;11:62. doi: 10.1186/s13062-016-0165-y (PMC5117590; doi:10.1186/s13062-016-0165-y)

A

Primer set	cDNA dilution	Log dilution	Average CT
ZFAS1-1	1/20	-1.30	20.43
	1/40	-1.60	20.88
	1/80	-1.90	22.00
	1/160	-2.20	23.15
	**Slope**	**-3.07**	
	**Efficiency**	**1.98**	

Primer set	cDNA dilution	Log dilution	Average CT
ZNFX1-1	1/20	-1.30	22.45
	1/40	-1.60	23.42
	1/80	-1.90	24.30
	1/160	-2.20	25.45
	**Slope**	**-3.28**	
	**Efficiency**	**1.97**	

Primer set	cDNA dilution	Log dilution	Average CT
ZFAS1-2	1/20	-1.30	22.44
	1/40	-1.60	22.98
	1/80	-1.90	23.94
	1/160	-2.20	24.93
	**Slope**	**-2.79**	
	**Efficiency**	**2.28**	

Primer set	cDNA dilution	Log dilution	Average CT
ZNFX1-2	1/20	-1.30	24.45
	1/40	-1.60	24.66
	1/80	-1.90	25.45
	1/160	-2.20	26.55
	**Slope**	**-2.353487228**	
	**Efficiency**	**2.660121004**	

B

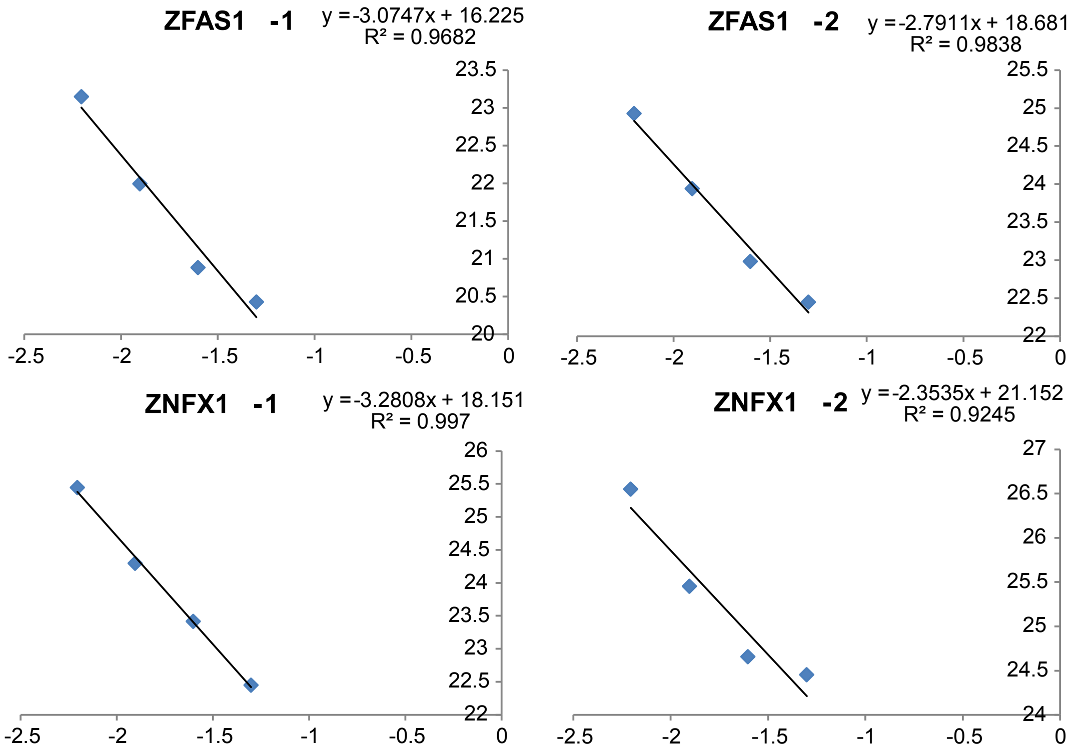

C

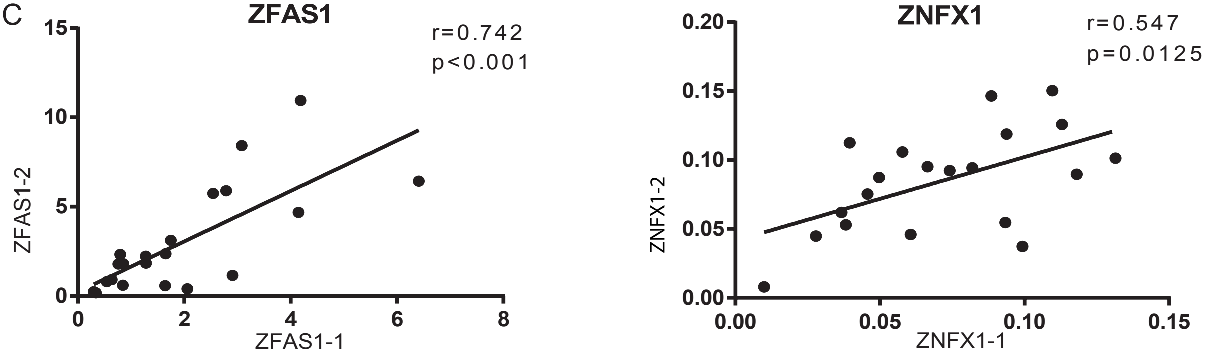

Supplement: Additional file 13: Figure S10. — Primer efficiency of ZFAS1 and ZNFX1. A) The primer efficiency test for the primers used in the experiments. B) Slopes of standard curve indicate PCR efficiency for ZFAS1 and ZNFX1 primers sets. The X axis represent the log of dilution. Y axis shows Ct values. C) Correlation between the expression of ZFAS1 and ZNFX1 in two different primer sets in panel of cell lines (PDF 317 kb) [file 13062_2016_165_MOESM13_ESM.pdf]
